# P2X_7_ receptors exhibit at least three modes of allosteric antagonism

**DOI:** 10.1126/sciadv.ado5084

**Published:** 2024-10-04

**Authors:** Adam C. Oken, Ismayn A. Ditter, Nicolas E. Lisi, Ipsita Krishnamurthy, Michael H. Godsey, Steven E. Mansoor

**Affiliations:** ^1^ Department of Chemical Physiology and Biochemistry, Oregon Health & Science University, Portland, OR 97239, USA.; ^2^ Division of Cardiovascular Medicine, Knight Cardiovascular Institute, Oregon Health & Science University, Portland, OR 97239, USA.

## Abstract

P2X receptors are trimeric ion channels activated by adenosine triphosphate (ATP) that contribute to pathophysiological processes ranging from asthma to neuropathic pain and neurodegeneration. A number of small-molecule antagonists have been identified for these important pharmaceutical targets. However, the molecular pharmacology of P2X receptors is poorly understood because of the chemically disparate nature of antagonists and their differential actions on the seven constituent subtypes. Here, we report high-resolution cryo–electron microscopy structures of the homomeric rat P2X_7_ receptor bound to five previously known small-molecule allosteric antagonists and a sixth antagonist that we identify. Our structural, biophysical, and electrophysiological data define the molecular determinants of allosteric antagonism in this pharmacologically relevant receptor, revealing three distinct classes of antagonists that we call shallow, deep, and starfish. Starfish binders, exemplified by the previously unidentified antagonist methyl blue, represent a unique class of inhibitors with distinct functional properties that could be exploited to develop potent P2X_7_ ligands with substantial clinical impact.

## Teaser

A P2X_7_ inhibitor defines the extended allosteric binding site, allowing the categorization of antagonists into three classes.

## INTRODUCTION

P2X receptors (P2XRs) are trimeric, nonselective cation channels activated by adenosine triphosphate (ATP) that act as molecular sensors for extracellular ATP (eATP) (*1*–*8*). P2XRs are distributed throughout the body as homomeric and heteromeric assemblies of three subunits from the P2X_1_–P2X_7_ gene family (*3*, *6*, *9*, *10*). The subunit composition of P2XRs determines their activation and desensitization kinetics in response to the binding of eATP (*3*, *6*, *9*–*16*). Because of their broad distribution and divergent physiological roles, P2XR dysfunction contributes to a wide variety of human diseases, including hypertension, hearing loss, asthma, and neuropathic pain (*6*, *11*, *17*–*27*). P2XRs are, therefore, important targets for therapeutic intervention (*6*, *20*, *28*, *29*).

Predominantly expressed in immune cells, homomeric P2X_7_ receptors are molecular sensors for pathological inflammatory states by detecting and responding to mid-micromolar to low-millimolar local concentrations of eATP (*16*, *22*, *30*, *31*). P2X_7_ activation triggers signaling cascades that result in up-regulation of the NLRP3 (NOD-, LRR-, and pyrin domain–containing protein 3) inflammasome and activation of apoptotic pathways (*32*–*38*). These cellular responses contribute to cardiovascular, neural, and immune diseases such as atherosclerosis, Alzheimer’s disease, and cancer (*22*, *24*, *39*–*43*). Such a broad influence on inflammatory processes renders the P2X_7_ receptor subtype a particularly enticing drug target.

For these reasons, there is an urgent need to identify selective and potent modulators of specific P2XR subtypes. Some subtype-specific small-molecule allosteric antagonists have been reported, providing insight into the molecular scaffolds and chemical compositions that contribute to pharmacological diversity (*30*). For instance, the P2X_3_-selective inhibitor AF-219 is structurally different from the P2X_4_-selective antagonist 5-BDBD or the P2X_7_-selective antagonist JNJ47965567 (fig. S1) (*30*). However, substantial chemical diversity also exists between antagonists that target the same P2XR subtype, including the P2X_7_-specific antagonists Brilliant Blue G (BBG) and A438079, which vary in size and shape as well as stereochemical and electrostatic properties (fig. S1) (*25*, *30*, *44*). Thus, the molecular blueprint for developing subtype-selective antagonists has remained elusive.

**Fig. 1.**
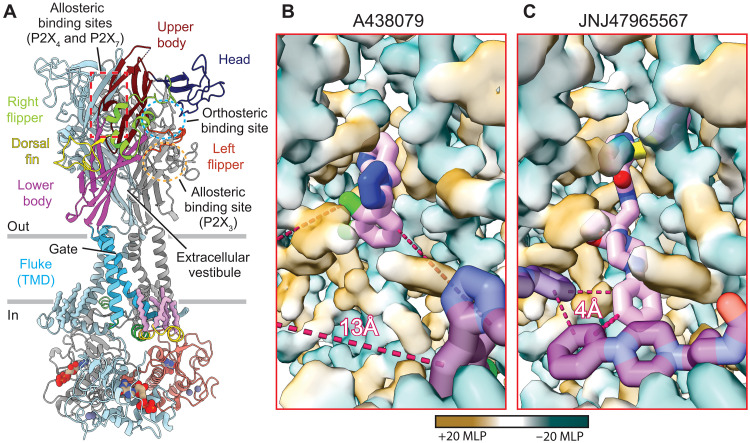
The classical allosteric rP2X_7_ ligand-binding site is hydrophobic. (**A**) Ribbon representation of the apo closed-state structure of rP2X_7_ (Protein Data Bank code: 8TR5) (*48*). One protomer is colored and labeled by domain architecture as previously described, and the other two protomers are colored gray and light blue (*11*, *16*, *65*). The classical allosteric ligand-binding site found in P2X_7_ is boxed in red and the orthosteric ATP-binding site is circled in blue. Because of the structural conservation of P2XRs, the known allosteric sites of human P2X_3_ (circled in yellow) and zebrafish P2X_4_ (boxed in red) are mapped to their respective spatial positions on rP2X_7_ (*15*, *45*). (**B** and **C**) Hydrophobic surface renderings of A438079-bound (B) and JNJ47965567-bound (C) rP2X_7_. Hydrophobic regions are colored brown [positive molecular lipophilicity potential (MLP)], and hydrophilic regions are colored turquoise (negative MLP). Surface renderings for A438079-bound and JNJ47965567-bound rP2X_7_ were generated at the same resolution as their cryo-EM reconstructions (2.2 and 2.4 Å, respectively). (B) Two symmetry-related molecules of A438079 (light pink and purple) are shown. The third molecule is out of frame due to 13-Å inter-ligand distances (dashed red lines). (C) Three symmetry-related molecules of JNJ47965567 (light pink, purple, and dark purple) are each separated by 4-Å inter-ligand distances (dashed red lines).

A structural understanding of allosteric binding pockets in different P2XRs is crucial for guiding the development of subtype-selective inhibitors. Extracellular allosteric sites have been described at the molecular level for three P2XR subtypes (*45*–*47*). An allosteric pocket has been identified just below the orthosteric ATP-binding site in human P2X_3_ (Fig. 1A) (*46*). Moreover, a locationally distinct allosteric ligand-binding site has been characterized in the upper body domain of zebrafish P2X_4_ and panda P2X_7_ (pdP2X_7_) (Fig. 1A) (*45*, *47*). This site in P2X_7_ will be referred to as the classical allosteric ligand-binding site. However, the pdP2X_7_ construct used for crystallization had truncated amino- and carboxy-terminal residues and an absent cytoplasmic domain, resulting in non-wild-type ion channel function (*47*). Furthermore, all existing structures of allosteric antagonist-bound P2XRs are at resolutions that limit the molecular description of ligand-receptor interactions (*45*–*47*).

We overcome these limitations by using structural, biophysical, and electrophysiological methods to define the molecular details of allosteric antagonism in full-length, wild-type rat P2X_7_ (rP2X_7_) receptors at high resolution. Our cryo-electron microscopy (cryo-EM) structures of six chemically diverse noncompetitive antagonists bound to rP2X_7_ reveal the ligand-receptor and inter-ligand interactions that underlie three functionally distinct classes of allosteric antagonists. Critically, we identify a P2X_7_ allosteric antagonist that occupies a previously uncharacterized binding site that we refer to as the extended allosteric ligand-binding site. Ligands bind to three distinct pockets within this site and form inter-ligand interactions that result in unique pharmacological properties. Together, our data provide vital details that will empower structure-based drug design of innovative P2X_7_-modulating ligands.

## RESULTS

### Chemically diverse antagonists bind to the same hydrophobic region of P2X_7_

To understand the molecular complexity of allosteric modulation of P2X_7_ receptors_,_ we used cryo-EM to obtain high-resolution structures of full-length, wild-type rP2X_7_ in multiple antagonist-bound inhibited states (figs. S2 and S3 and table S1). In each case, we observed a molecule of antagonist bound to each subunit in the trimeric receptor, and the resolutions between 2.2 and 2.7 Å afforded detailed visualization of ligand-protein interactions and non-proteinaceous features (table S1). For example, densities for water molecules are readily observed throughout the receptor, including key areas surrounding the ligand-binding pockets. In addition, in structures with resolutions better than ~2.5 Å, features and topology of the P2X_7_ pore, as previously described, become clearly resolved (*48*). First, a partially hydrated Na^+^ ion is present in the center of the closed pore, just above the gate, interacting with three symmetrically located water molecules and S342 in each protomer (fig. S4A) (*48*). This finding is also present in reconstructions processed without imposed symmetry (fig. S4B). Second, water molecules located above and below the gate stabilize a short 3_10_-helical region of transmembrane helix 2 (residues 340 to 344) (*48*). Last, intracellular water molecules found just below the gate, and coordinated by S342 from each protomer, are ideally poised to rehydrate ions passing through the open channel (fig. S4A). Together, these components appear necessary for properly positioning residues that form the gate in the antagonist-bound inhibited state (*48*).

Five of our structures provide insight into the binding of known P2X_7_ allosteric antagonists at atomic resolution: A438079 (2.2 Å), A839977 (2.5 Å), AZD9056 (2.2 Å), GSK1482160 (2.4 Å), and JNJ47965567 (2.4 Å) (figs. S1 to S3 and table S1). These chemically diverse antagonists bind to an extracellular site identified in a previous crystallographic study of a truncated pdP2X_7_ construct, which we refer to as the classical allosteric ligand-binding site (Fig. 1A and fig. S5A) (*47*). Our improved resolutions provide the molecular details of ligand binding for each antagonist (fig. S3 and table S1), and the full-length, wild-type construct allows visualization of the complete transmembrane and cytoplasmic domains (Fig. 1A and fig. S5B). The structures show that the transmembrane and cytoplasmic domains of rP2X_7_ are unaffected by allosteric antagonist binding (fig. S5). However, key portions of the extracellular domain, including loop residues 88 to 100 in the upper body, move to accommodate the distinct scaffolds and receptor interactions made by these ligands (Fig. 1A and figs. S5 and S6, A and B).

### Allosteric antagonists form inter-ligand and ligand-receptor interactions

**Fig. 2.**
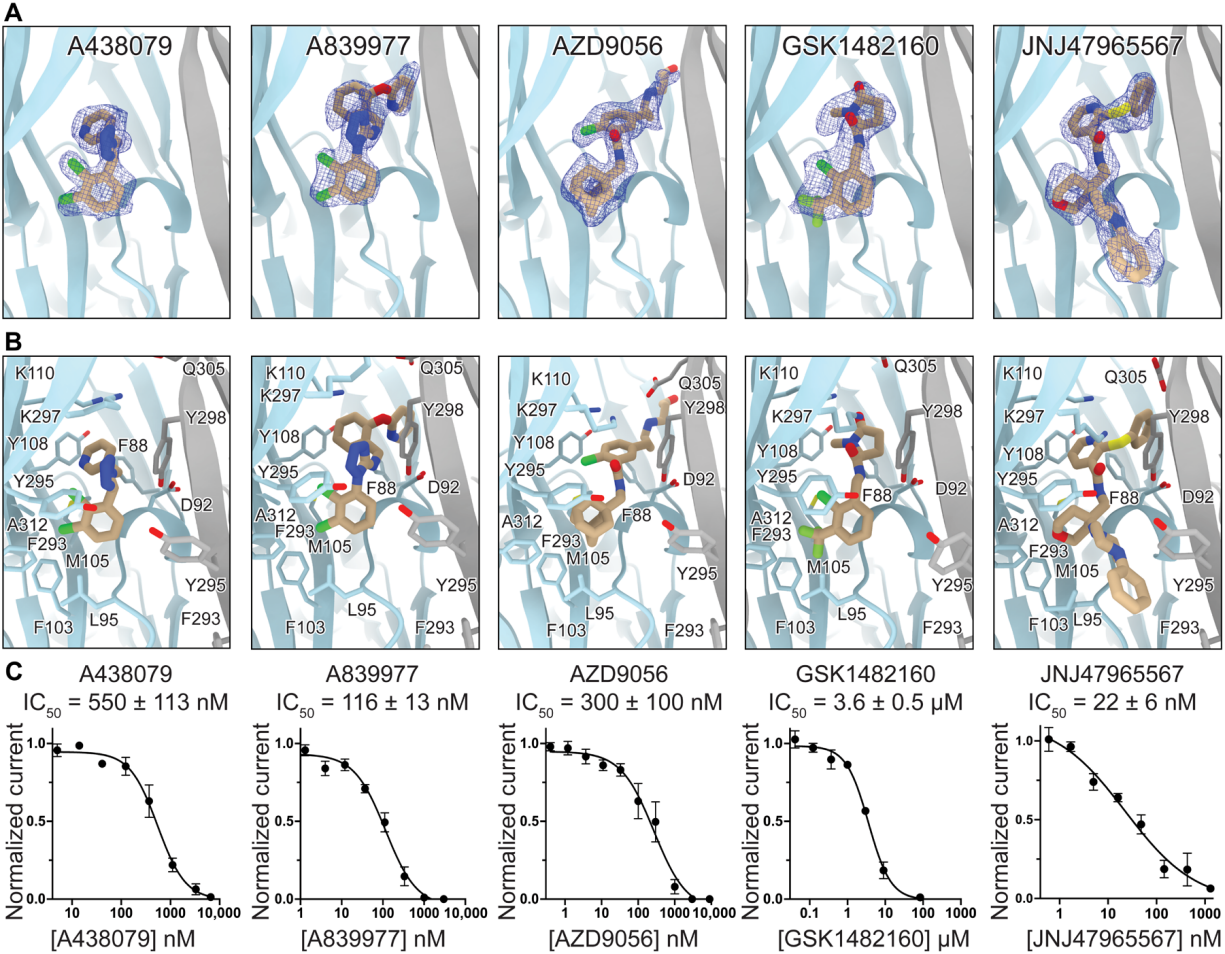
Antagonists bind to rP2X_7_ at the classical allosteric ligand-binding site. (**A**) Ribbon representation of the classical allosteric rP2X_7_ ligand-binding site at the interface of two protomers (gray and light blue) bound to one molecule (left to right) of A438079 (2.2 Å), A839977 (2.5 Å), AZD9056 (2.2 Å), GSK1482160 (2.4 Å), and JNJ47965567 (2.4 Å) with corresponding electron density for each ligand (blue mesh). (**B**) Residues in the classical allosteric ligand-binding site that interact with (left to right) A438079, A839977, AZD9056, GSK1482160, and JNJ47965567. F103 only participates in hydrophobic interactions with AZD9056, GSK1482160, and JNJ47965567. Toward the surface of the binding site, W167 forms hydrophobic interactions with GSK1482160 and JNJ47965567. The halogen atoms in A438079, A839977, and GSK1482160 all point toward A312. K110 forms cation-pi interactions with A839977 and AZD9056 and hydrogen bonds with GSK1482160 (distance of 2.9 Å). (**C**) Inhibition dose-response (IC_50_) curves measured by two-electrode voltage clamp (TEVC) for (left to right) A438079, A839977, AZD9056, GSK1482160, and JNJ47965567. Data points and error bars represent means and SDs of normalized current across triplicate experiments, respectively.

The resolutions achieved in our reconstructions allowed unambiguous placement of the various antagonists within the cryo-EM densities (Fig. 2A and fig. S7), revealing key ligand-receptor interactions within the classical allosteric ligand-binding site (Fig. 2B). The structure of A438079-bound rP2X_7_ reveals the noncompetitive nature of this ligand, in contrast to previous data (*49*). Although polar residues are found at the entrance to this binding site, located near the top of the extracellular domain, the majority of the site is composed of hydrophobic residues (Figs. 1, B and C, and 2B). In all five allosteric antagonist-bound rP2X_7_ structures, the conserved hydrophobic pocket is lined by residues F88, L95, F103, M105, Y108, F293, Y295, Y298, I310, and A312 (Fig. 2B and figs. S8 and S9). Each of these residues participates in hydrophobic interactions with the five antagonists, although we observed conformational differences across the various structures. Mutation of F88 or F103 to alanine generally results in decreased inhibitory potencies (table S2). These residues are in analogous positions to those that form the classical allosteric ligand-binding site and affect inhibitory potency in pdP2X_7_ (*47*). Ligands bind to this site and prevent the narrowing of the classical allosteric pocket that occurs upon ATP-binding, the so-called “turret” motion, which is necessary for transition to the ATP-bound open state of P2X_7_ (*47*). In our JNJ47965567-bound structure, the three molecules of antagonist extend deep enough into each classical allosteric ligand-binding site to reach within ~4 Å of each other and create edge-to-edge inter-ligand hydrophobic interactions (Fig. 1C). In contrast, all other allosteric antagonists bind more shallowly, situating them ~11 to 14 Å from their symmetry-related molecules and preventing any inter-ligand interactions (Fig. 1B).

In addition to the hydrophobic interactions described above, D92 and K297 in rP2X_7_ form hydrogen bonds with atoms in the middle of several of the antagonists (Fig. 2B and figs. S8 and S9). The backbone carbonyl of D92 forms hydrogen bonds with the amide nitrogens of AZD9056, GSK1482160, and JNJ47965567 (at distances of 2.9, 3.2, and 2.7 Å, respectively) as well as a hydrogen bond with the tetrazole ring of A839977 (at a distance of 3.1 Å) (Fig. 2B and figs. S1, S8, and S9). This latter hydrogen bond is not present in the A438079-bound structure as this ligand lacks an appropriately placed nitrogen or functional group (Fig. 2B and fig. S1). The ammonium group in K297 participates in hydrogen bonds with the amide carbonyl on GSK1482160 and JNJ47965567 (at distances of 2.9 and 2.8 Å, respectively) (Fig. 2B and figs. S1, S8, and S9). These ligand-receptor interactions involving D92 and K297 were not apparent in the lower-resolution structures of pdP2X_7_ (*47*). Unexpectedly, a K297V mutation increases or does not affect the inhibitory potency of the antagonists (table S2). While removing a seemingly important hydrogen bonding interaction, the mutation likely creates additional hydrophobic interactions that compensate. A similar observation was made for a K297G mutation (*50*, *51*).

Our cryo-EM reconstructions identified additional interactions, including the coordination of allosteric ligands by residues at the surface of the classical allosteric ligand-binding site, exemplified by the interaction between K110 and the oxygen on the pyroglutamide moiety of GSK1482160 (at a distance of 2.9 Å) (Fig. 2B and figs. S1 and S8). We also observed water molecules interacting with each of the ligands; an important consideration for structure-based drug design (*52*, *53*). Together, our high-resolution structures revealed interactions between the five chemically diverse allosteric antagonists and the classical allosteric ligand-binding site that likely contribute to the broad range of inhibitory potencies (IC_50_) observed for these ligands (Fig. 2C and table S2).

### Methyl blue forms extensive interactions in a unique mode of antagonism

**Fig. 3.**
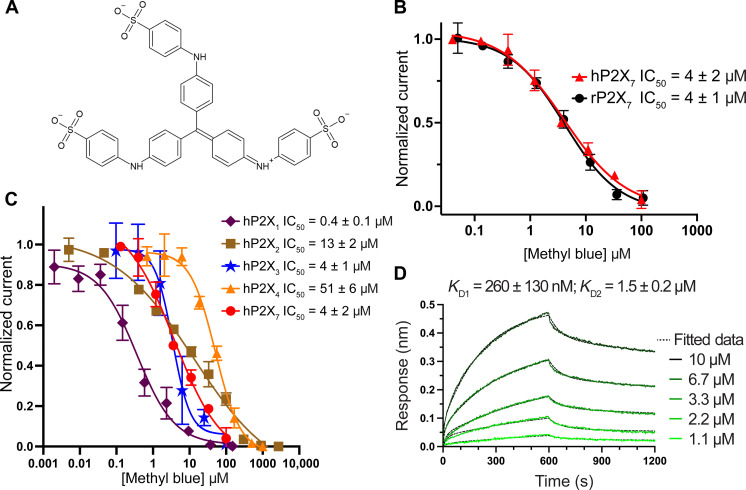
Methyl blue is an allosteric antagonist of P2X_7_. (**A**) Two-dimensional chemical structure of methyl blue, a symmetric analog of Brilliant Blue G, comprising three resonance-related diphenylamine-4-sulfonate arms. (**B**) Inhibition dose-response curves recorded by TEVC for methyl blue antagonism of rP2X_7_ (IC_50_ = 4 ± 1 μM) and hP2X_7_ (IC_50_ = 4 ± 2 μM). Data points and error bars represent means and SDs of normalized current across triplicate experiments, respectively. (**C**) Inhibition dose-response curves recorded by TEVC for methyl blue antagonism of hP2X_1_, hP2X_2_, hP2X_3_, hP2X_4_, and hP2X_7_. Data points and error bars represent means and SDs of normalized current across triplicate experiments, respectively. (**D**) Representative BLI sensorgram for a methyl blue dilution series binding to biotinylated rP2X_7_ immobilized on streptavidin biosensors during a 600-s association time and a 600-s dissociation time. Data were fit with a 2:1 Langmuir model with rate constants for association (*k*_a_) of *k*_a1_ = 4.5 ± 0.2 × 10^2^ M^−1^ s^−1^ and *k*_a2_ = 9.3 ± 2.3 × 10^3^ M^−1^ s^−1^ and rate constants for dissociation (*k*_d_) of *k*_d1_ = 1.1 ± 0.5 × 10^−4^ s^−1^ and *k*_d2_ = 1.3 ± 0.1 × 10^−2^ s^−1^, respectively. This resulted in a two-component equilibrium disassociation constant (*K*_D_) for methyl blue binding with a high-affinity component (*K*_D1_ = 260 ± 130 nM; 67 ± 8% of signal) and a low-affinity component (*K*_D2_ = 1.5 ± 0.2 μM; 33 ± 8% of signal). Kinetic values represent mean and SD across triplicate experiments.

BBG is an established allosteric P2X_7_-selective antagonist with a large scaffold (fig. S1) (*44*). To investigate how such a large molecule could bind to the classical allosteric ligand-binding site, we searched for suitable analogs of BBG that act on P2X_7_ receptors. Two-electrode voltage-clamp (TEVC) recordings identified methyl blue as an effective antagonist of both rP2X_7_ (IC_50_ = 4 ± 1 μM) and human P2X_7_ (hP2X_7_) (IC_50_ = 4 ± 2 μM) (Fig. 3, A and B, and fig. S1). In addition, the selectivity of methyl blue was tested across P2XR subtypes showing that it is a rather non-specific P2XR antagonist with potencies ranging between 0.4 ± 0.1 μM for hP2X_1_ and 51 ± 6 μM for hP2X_4_ (Fig. 3C). The kinetics and equilibrium binding affinity of methyl blue on rP2X_7_ were measured by bio-layer interferometry (BLI) and fitted to a 2:1 Langmuir model, revealing a high-affinity binding component (*K*_D1_ = 260 ± 130 nM; 67 ± 8% of signal) and a low-affinity binding component (*K*_D2_ = 1.5 ± 0.2 μM; 33 ± 8% of signal) (Fig. 3D). Unlike the pseudo-symmetric BBG molecule, methyl blue has threefold symmetry, in which each arm of the scaffold contains a resonance-related diphenylamine-4-sulfonate moiety (Fig. 3A and fig. S1).

**Fig. 4.**
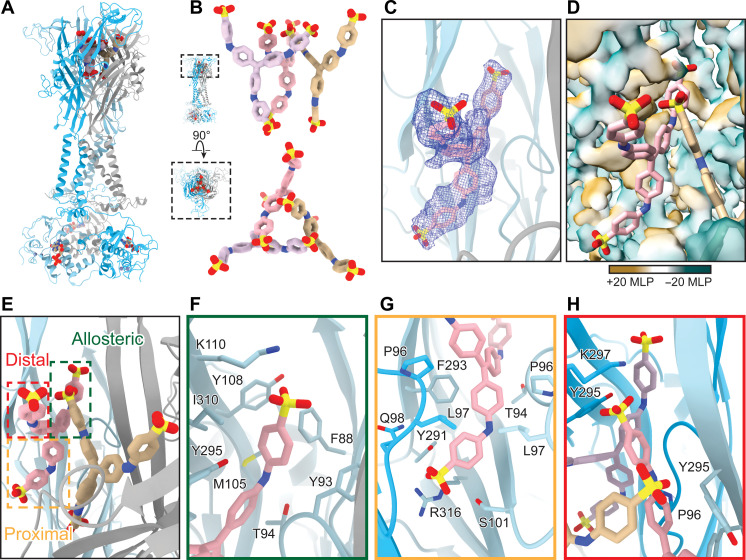
Methyl blue binds to an extended allosteric ligand-binding site. (**A**) Ribbon representation of the methyl blue–bound inhibited state structure of rP2X_7_ at 2.7 Å. Protomers are represented by gray, light blue, and blue. Three molecules of methyl blue bind to the receptor, each at one of the three symmetry-related extended allosteric ligand-binding sites. (**B**) Orthogonal views (top, side-on; bottom, top-down) highlight the unique binding mode and inter-ligand interactions between different molecules of methyl blue (tan, light pink, and light purple). (**C** to **E**) Identical views of methyl blue bound at the extended allosteric ligand-binding site of rP2X_7_. (C) Extended allosteric rP2X_7_ ligand-binding site showing only one molecule of methyl blue and its corresponding electron density (blue mesh). (D) Hydrophobicity of the extended allosteric ligand-binding site of rP2X_7_, represented as a transparent 2.7-Å resolution surface rendering, with only two molecules of methyl blue shown (represented in light pink and tan). Hydrophobic regions are colored brown (positive MLP) and hydrophilic regions are colored turquoise (negative MLP). (E) Methyl blue bound in the extended allosteric ligand-binding site, with the allosteric, proximal, and distal arms of the molecule boxed in green, yellow, and red, respectively. (**F**) Residues that interact with the allosteric arm of methyl blue (F88, Y93, T94, M105, Y108, K110, F293, Y295, and I310) define the classical allosteric pocket. (**G**) Residues that interact with the proximal arm (relative to the center of the receptor) of methyl blue (T94, P96, L97, Q98, G99, S101, Y291, F293, and R316) define the proximal pocket. (**H**) Residues that interact with the distal arm of methyl blue (P96, Y295, and K297) define the distal pocket [view rotated by 90° from (E)]. The distal arm of each methyl blue molecule (tan, light pink, and light purple) forms inter-ligand interactions between symmetry-related molecules.

Our high-resolution (2.7 Å) cryo-EM reconstruction of methyl blue bound to full-length, wild-type rP2X_7_ reveals how this ligand occupies a unique allosteric ligand-binding site that we term the extended allosteric ligand-binding site (Fig. 4 and figs. S2 and S3 and table S1). Similar to other ligand-bound structures of P2X_7_, three molecules of methyl blue bind to the trimeric receptor with C3 symmetry (Fig. 4, A and B) (*47*). However, there are critical differences in how methyl blue occupies the extended allosteric ligand-binding site that make the molecular pharmacology of this interaction distinct (Fig. 4, C to H). The head domain of the receptor expands (residues 110 to 173) and three sets of loop residues 70 to 85, 88 to 100, and 296 to 308 are uniquely displaced to accommodate the binding of the large ligand (Fig. 1A and figs. S5 and S6, C and D). Moreover, each of the three diphenylamine-4-sulfonate arms of a single methyl blue molecule splays out to occupy a different pocket within the extended allosteric ligand-binding site (Fig. 4, E to H).

One arm of methyl blue occupies the classical allosteric ligand-binding site, interacting with many of the same residues as the smaller ligands, including F88, Y93, T94, M105, Y108, K110, F293, Y295, and I310 (Figs. 2B and 4, E and F). This “allosteric arm” is coordinated in the classical allosteric pocket by a combination of polar and nonpolar interactions. K110 forms two hydrogen bonds with oxygens on the sulfonate group (at distances of 3.1 and 3.5 Å) (Fig. 4F). In addition, the hydroxyl of Y295 and backbone carbonyl of Y93 form hydrogen bonds with the nitrogen within this arm of methyl blue (at distances of 3.5 and 3.4 Å, respectively) (Fig. 4F).

A second arm of methyl blue, identified as a “proximal arm” relative to the center of the receptor, extends deep into the protein adjacent to the axis of symmetry in a pocket surrounded by the lower body domain and just above the extracellular vestibules (Figs. 1A and 4, E and G). The proximal arm interacts with residues T94, P96, L97, Q98, G99, S101, Y291, F293, and R316 in this proximal pocket (Fig. 4G), forming mainly hydrophobic interactions. In addition, the backbone nitrogen of residues Q98 and G99 as well as the guanidino group of R316 likely form hydrogen bonds with the sulfonate group of this arm (at distances of 2.6 to 3.8 Å) (Fig. 4G).

The third arm of methyl blue, termed the “distal arm,” extends toward the top of the extracellular domain to interact with the upper body and head domains of P2X_7_ near the axis of symmetry (Figs. 1A and 4, E and H). The residues in this distal pocket that interact with methyl blue include P96, Y295, and K297 (Fig. 4H). K297 forms hydrogen bonds with the sulfonate of the distal arm (at a distance of 3.0 Å) (Fig. 4H). Notably, each distal arm in the three molecules of methyl blue interacts with the distal arm of an adjacent, symmetry-related molecule, creating inter-ligand interactions (Fig. 4, B, E, and H). Clear edge-to-face interactions form between the distal arms of each methyl blue at the center of the receptor (at distances of ~4 Å) (Fig. 4H). This distinct binding mode of methyl blue, involving inter-ligand interactions and previously unappreciated binding pockets within an extended allosteric ligand-binding site, defines a framework to develop P2X_7_ antagonists with extensive interaction networks (Fig. 4).

### Three modes of binding are reflected in functional antagonistic properties

**Fig. 5.**
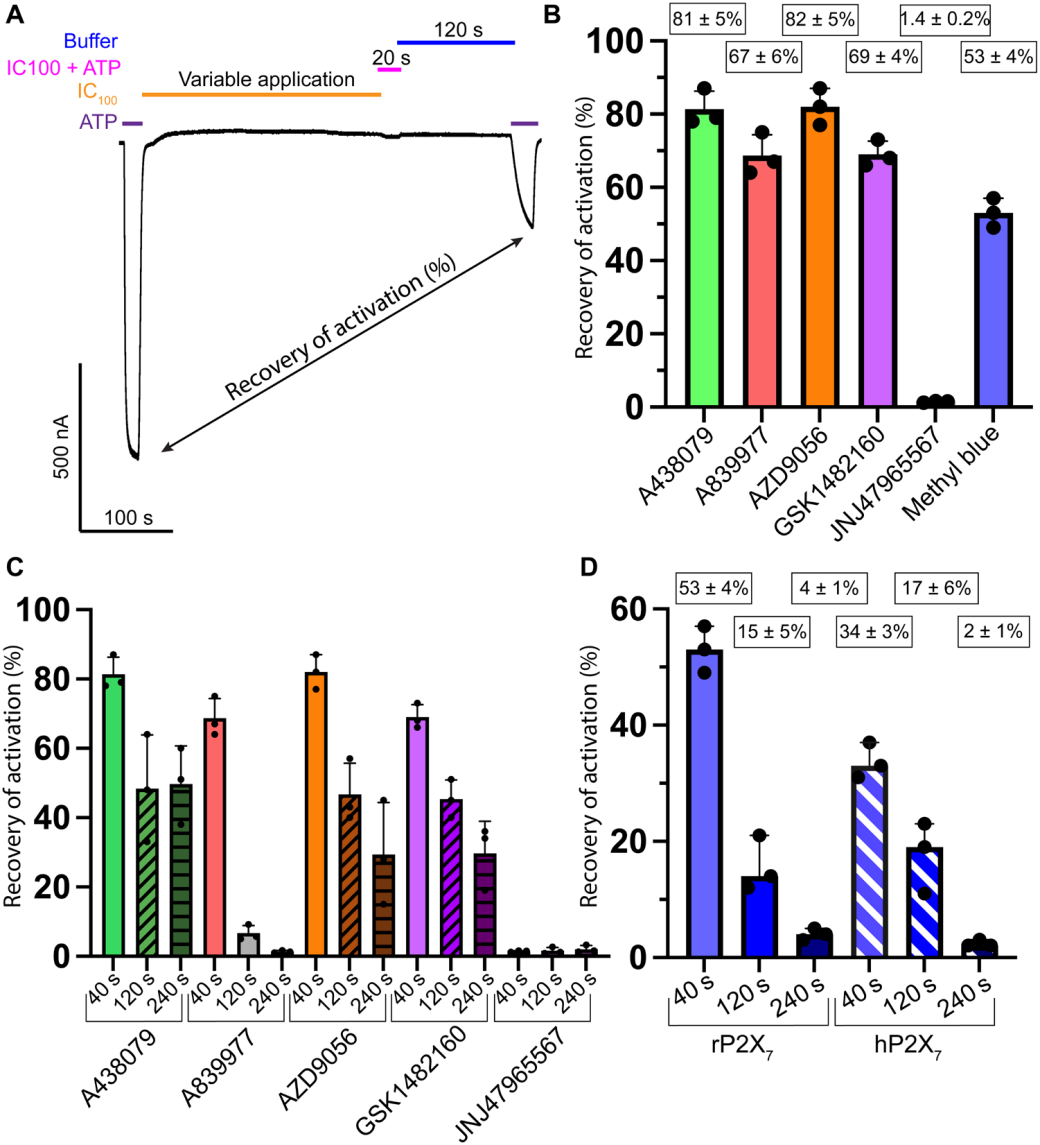
Application of methyl blue results in low recovery of activation following maximal antagonism. Recovery of P2X_7_ receptor activation expressed as a percentage of baseline current in response to 100 μM ATP following variable IC_100_ application times of antagonist and a fixed buffer wash. (**A**) Example TEVC trace for a recovery experiment. The percent recovery is the ratio of inward current evoked from a 100 μM ATP application before and after an IC_100_ application of antagonist for 40, 120, or 240 s. The experiment evaluates the relative rate of association and dissociation of each ligand to and from the receptor, respectively. (**B**) Recovery of activation after 40-s applications of the six allosteric antagonists tested. Antagonists A438079, A839977, AZD9056, and GSK1482160 permit strong receptor recovery; JNJ47965567 permits negligible receptor recovery; methyl blue permits moderate receptor recovery. (**C**) Recovery of activation following progressively longer IC_100_ applications of antagonists (40, 120, and 240 s). A438079, AZD9056, and GSK1482160 permit strong recovery even after long application of antagonist; JNJ47965567 permits negligible receptor recovery; and A839977 permits strong recovery after 40 s, but negligible recovery after longer applications. (**D**) Recovery of activation following progressively longer IC_100_ applications of methyl blue at rP2X_7_ and hP2X_7_. Channel recovery decreases with longer exposures to methyl blue until there is essentially no recovery after a 240-s application. Data represent means and SDs across triplicate experiments.

To investigate how effectively the various antagonists remain bound to rP2X_7_, we used TEVC to quantify the relative amount of ATP-induced channel activity following recovery from 40-, 120-, and 240-s exposures to 100% inhibitory concentrations (IC_100_) of antagonists (Fig. 5 and table S3). This experiment uses electrophysiology to evaluate the relative rates of association and dissociation of each ligand to and from the receptor, respectively. Following a 40-s exposure to an IC_100_ of antagonist, channel activity recovered more with exposure to AZD9056 and A438079 (82 ± 5% and 81 ± 5% of baseline current, respectively) when compared with exposure to A839977 and GSK1482160 (67 ± 6% and 69 ± 4% of baseline current, respectively) (Fig. 5B and table S3). These data indicate that A839977 and GSK1482160 more easily gain entry to the classical allosteric ligand-binding site and stay bound longer than AZD9056 and A438079, which more readily leave the binding pocket. After longer exposures to an IC_100_ of each antagonist (120 and 240 s), channel activity recovered less than following a 40-s exposure. While A438079, AZD9056, and GSK1482160 demonstrated progressively less recovery after longer IC_100_ exposure times, none of these antagonists completely prevented channel recovery (Fig. 5C and table S3). On the other hand, A839977 nearly completely prevented channel recovery after an IC_100_ exposure of 240 s (1.4 ± 0.3% of baseline current) (Fig. 5C and table S3).

Exposure to methyl blue prevents the recovery of channel activity more than might be expected for its modest inhibitory potency (Figs. 3B and 5, B and D, and table S3). Following increasingly longer IC_100_ applications of methyl blue (40, 120, and 240 s), rP2X_7_ activity recovered to 53 ± 4%, 15 ± 5%, and 4 ± 1% of baseline current, respectively (Fig. 5D and table S3). Similarly, hP2X_7_ activity only recovered to 34 ± 3%, 17 ± 6%, and 2 ± 1% of baseline current after longer applications of methyl blue (Fig. 5D and table S3). Furthermore, JNJ47965567—the most potent ligand tested—was most effective at preventing channel recovery across all timescales (only 1.4 ± 0.2%, 1.6 ± 1%, and 2 ± 1% of baseline current for 40-, 120-, and 240-s IC_100_ applications, respectively) and therefore most likely to remain bound to the classical allosteric ligand-binding site (Fig. 5, B and C, and table S3). These data suggest that there might be differences between ligand access to and exit from the respective binding sites that reflect different modes of binding.

## DISCUSSION

Our structural and functional data amassed from full-length, wild-type rP2X_7_ in six antagonist-bound inhibited states provide substantial insight into the molecular pharmacology of this clinically relevant P2XR subtype. The high-resolution structures of five chemically diverse allosteric antagonists bound to the classical allosteric ligand-binding site show that different ligand scaffolds and chemical moieties interact with this binding pocket at differing depths. Furthermore, the structure of the receptor in complex with the allosteric antagonist, methyl blue, reveals both an extended allosteric ligand-binding site and a distinct binding mode for antagonists. We thus group allosteric P2X_7_ antagonists into three classes that we call shallow, deep, and starfish binders, providing insight that could aid the development of treatments for the numerous pathophysiological conditions that result from P2X_7_ activity (*11*, *20*, *54*, *55*).

**Fig. 6.**
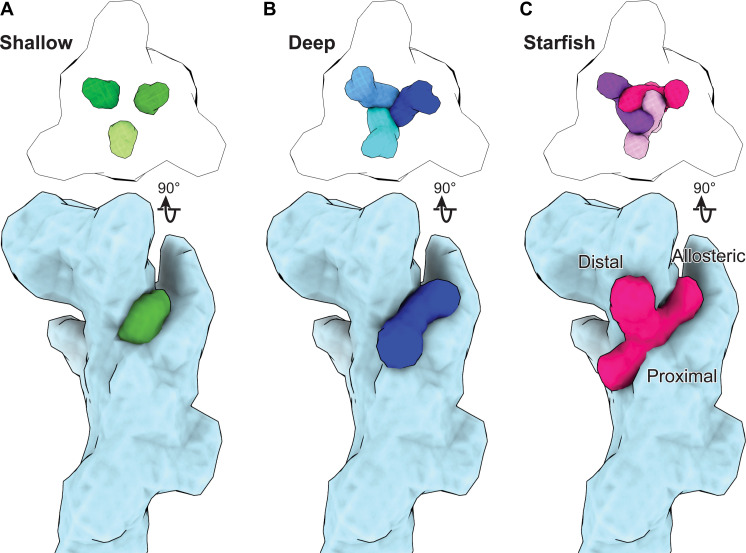
Three modes of allosteric antagonism of the P2X_7_ receptor. (**A** to **C**) Schematic representations of the distinct binding modes for three classes of allosteric antagonists bound to rP2X_7_ created using low-resolution synthetic maps generated from structural models. Top: Top-down view of the relative positions of the three symmetry-related ligands for each class of allosteric P2X_7_ antagonist. Bottom: Side view (rotated 90° from the top row) for the three classes of allosteric ligands bound to a single rP2X_7_ protomer. (A) Shallow binder A438079 occupies a shallow position in the classical allosteric ligand-binding site. Each ligand is located far from the other two symmetry-related molecules, preventing inter-ligand interactions. (B) Deep binder JNJ47965567 occupies a deep position in the classical allosteric ligand-binding site. Each ligand participates in edge-to-edge inter-ligand interactions with the other two symmetry-related molecules. (C) Starfish binder methyl blue occupies the extended allosteric ligand-binding site. The three binding arms of methyl blue are named allosteric, distal, and proximal according to the binding pocket for which each arm occupies (classical allosteric pocket, distal pocket, and proximal pocket). The distal arm of each starfish binder participates in extensive edge-to-face inter-ligand interactions with the distal arms of the other two symmetry-related molecules.

Our molecular characterization of allosteric P2X_7_ antagonists into three different binding modes is based on how each ligand binds to the receptor (Fig. 6). Shallow binders, represented by A438079, A839977, AZD9056, and GSK1482160, extend minimally into the classical allosteric ligand-binding site (Fig. 6A). Deep binders, represented by JNJ47965567, access deeper residues in the binding pocket well beyond those that interact with shallow binders (Fig. 6B). Starfish binders, represented by methyl blue, bind to a previously unappreciated extended allosteric ligand–binding site composed of the classical allosteric pocket in addition to proximal and distal pockets (Fig. 6C). The volume of the pocket in the classical allosteric ligand-binding site is moderately decreased for zfP2X_4_ and even more substantially for hP2X_3_ (fig. S10) (*15*, *45*). These structural differences can be leveraged by designing chemical moieties on P2XR antagonists to match receptor pockets, thereby tuning subtype-specificity.

We observed several specific interactions between shallow binding ligands and residues D92, K297, Y298, and K110 that we speculate may affect the ability of the ligands to inhibit the receptor. The structures of P2X_7_ in complex with the tetrazole-based antagonists, A438079 and A839977, provide some initial insights. First, the nitrogen bonded to the tetrazole ring in A839977, which is absent in A438079, forms hydrogen bonds with D92 and Y298. Second, the additional rings found in A839977, but not A438079, participate in hydrophobic interactions at the entrance to the classical allosteric ligand-binding site. These two differences could possibly contribute to the slightly higher inhibitory potency of A839977 (IC_50_ = 116 ± 13 nM) compared to A438079 (IC_50_ = 550 ± 113 nM). While the importance of the backbone interaction with D92 is not testable using site-directed mutagenesis, mutation of Y298 led to nonfunctional channels as tested by TEVC (table S2). In chimeric P2X_7_ channels, a Y298A mutation was shown to generally decrease the potency of several antagonists (*50*, *51*). The adamantane-containing ligand AZD9056 is also coordinated by hydrophobic interactions and two hydrogen bonds with D92 and Y298, but not with K297. This, together with the increased hydrophobicity of AZD9056, could possibly explain its modest inhibitory potency (IC_50_ = 330 ± 100 nM). GSK1482160 is coordinated by D92, K110, K297, and Y298, but lacks hydrophobic moieties on either end of the ligand, resulting in a lower potency (IC_50_ = 3.6 ± 0.5 μM) than the other shallow binders. All shallow binding ligands are too far apart from their symmetrically located counterparts to participate in inter-ligand interactions (Figs. 1B and 6A).

The deep binder, JNJ47965567, is structurally and functionally different than shallow binders. It extends deeper into the classical allosteric ligand-binding site, enabling additional interactions with the receptor (Fig. 6B). Furthermore, each JNJ47965567 molecule is close enough to its symmetrically located counterparts to participate in edge-to-edge interactions (Figs. 1C and 6B). As a result of this extensive coordination, inter-ligand interactions, and deep penetration into the binding site, JNJ47965567 is the most potent of the antagonists tested (IC_50_ = 22 ± 6 nM) and results in minimal recovery of channel activity after antagonism at all time points (recovery after IC_100_ of only 1.4 ± 0.2%, 1.6 ± 1%, and 2 ± 1% for 40, 120, and 240 s, respectively), two key properties that can be leveraged for structure-based drug design.

The previously unidentified starfish binder, methyl blue, expands our knowledge of P2X_7_ modulation by revealing the extended allosteric ligand-binding site (Fig. 6C). When bound to rP2X_7_, the three resonance-related diphenylamine-4-sulfonate moieties of methyl blue can be defined as proximal, distal, and allosteric arms that bind in proximal, distal, and classical allosteric pockets, respectively (Figs. 4, E to H, and 6C). Each arm participates in a unique set of interactions that contribute to an unexpectedly weak inhibitory potency (relative to shallow and deep binders) at both rP2X_7_ (IC_50_ = 4 ± 1 μM) and hP2X_7_ (IC_50_ = 4 ± 2 μM) likely due to an effect of steric bulk on the rate of association to the receptor. Methyl blue also has a slow rate of dissociation from the binding pocket, particularly after longer exposures, likely due to a vast network of ligand-receptor and inter-ligand interactions that lock the ligand in place once all three sites in the extended allosteric ligand-binding sites are occupied (Fig. 6C). Methyl blue blocks the receptor’s ability to recover channel activity more strongly than most shallow binders, despite having a lower apparent affinity, reinforcing the idea that this ligand leverages a different mode of binding (Figs. 2C, 3, and 5).

The rates of association and dissociation for methyl blue binding to rP2X_7_ were successfully fit to a 2:1 Langmuir binding model, but not a 1:1 model, revealing two different equilibrium dissociation constants (*K*_D_) for methyl blue molecules and therefore indicating some amount of cooperative binding. One molecule of methyl blue associates and dissociates quickly with low-affinity binding (*K*_D2_ = 1.5 ± 0.2 μM), explaining why shorter applications result in more recovery of channel activity after exposure to an IC_100_. In contrast, the other two molecules of methyl blue associate and disassociate much slower with high-affinity binding (*K*_D1_ = 260 ± 130 nM), explaining why longer applications of methyl blue more effectively hinder the ability of the receptor to recover from antagonism. This high-affinity binding of methyl blue is apparent in our structural data showing that the binding of three molecules is sterically challenging but that, once bound, they become locked into the receptor like an irreversible inhibitor. Thus, methyl blue exhibits cooperative ligand binding at the extended allosteric ligand-binding site of rP2X_7_, largely driven by its steric bulk and the ability of the receptor to expand to accommodate its large scaffold. These findings establish a framework to understand how ligands with large scaffolds can modulate P2X_7_ activity and develop potent and selective antagonists that can bind tightly to the receptor for long periods of time. In particular, the short inter-ligand contacts between molecules of deep and starfish binders introduce the intriguing possibility of designing multivalent antagonists, in which independent binding moieties chemically linked in a single molecule could increase affinity and specificity for P2X_7_, thereby decreasing unwanted off-target effects (*56*, *57*). Together, our data defines inter-ligand and ligand-receptor interactions across different chemical moieties, classes, and scaffolds for the P2X_7_ receptor, providing the molecular building blocks as the blueprints to develop highly potent and selective ligands with substantial potential to treat clinical diseases.

## MATERIALS AND METHODS

### Cell lines

SF9 cells of a female origin were cultured in SF-900 III SFM (Thermo Fisher Scientific) at 27°C and used for the expression of baculovirus. Human embryonic kidney (HEK) 293 GNTI^−^ cells of female origin were grown in Gibco Freestyle 293 Expression Medium (Thermo Fisher Scientific) at 37°C supplemented with 2% v/v fetal bovine serum and used to express recombinant protein (*58*). Ecocyte Bioscience was used to source unfertilized *Xenopus laevis* oocytes which were kept at 18°C until injection.

### Receptor constructs

Structural determination of the full-length, wild-type rP2X_7_ was completed using a construct previously described containing no truncations or mutations (*16*). Furthermore, the rP2X_7_-WT construct used for electrophysiology experiments is unmodified full-length, wild-type rP2X_7_ with no green fluorescent protein (GFP), protease sites, or affinity tags present. The hP2X_1_-WT, hP2X_2_-WT, hP2X_3_-WT, hP2X_4_-WT, and hP2X_7_-WT constructs used for electrophysiology are similarly unmodified full-length, wild-type receptor constructs with no GFP, protease sites, or affinity tags present. Mutations to the rP2X_7_-WT construct used for electrophysiology experiments were made directly in the pcDNA3.1x plasmid. These constructs included the following: rP2X_7_-K297A, rP2X_7_-K297G, rP2X_7_-K297M, rP2X_7_-K297Q, rP2X_7_-K297V, rP2X_7_-Y298A, rP2X_7_-Y298G, rP2X_7_-Y298V, rP2X_7_-Y298L, rP2X_7_-R316A, rP2X_7_-R316G, rP2X_7_-R316Q, rP2X_7_-R316H, rP2X_7_-F88A, rP2X_7_-F103A, and rP2X_7_-Y295A.

### Receptor expression and purification

Baculovirus-mediated gene transfection (BacMam) was used to express the full-length, wild-type rP2X_7_ construct, as previously outlined in identical protocols (*16*). Briefly, HEK293 GNTI^−^ cells were grown in suspension to a sufficient density and infected with the P2 BacMam virus. After overnight growth at 37°C, sodium butyrate was added (final concentration of 10 mM), and cells were shifted to 30°C for an additional 48 hours. The cells were then harvested by initially washing with phosphate-buffered saline (PBS) buffer (137 mM NaCl, 2.7 mM KCl, 8 mM Na_2_HPO_4_, and 2 mM KH_2_PO_4_), followed by suspension in TBS [50 mM tris (pH 8.0) and 150 mM NaCl] containing protease inhibitors [1 mM phenylmethylsulfonyl fluoride, aprotinin (0.05 mg/ml), pepstatin A (2 mg/ml), and leupeptin (2 mg/ml)], and lysis was performed via sonication. Centrifugation successfully separated intact cells and cellular debris from membranes that were then isolated by ultracentrifugation. All membranes were snap-frozen and stored at −80°C until use.

Thawed membranes were resuspended in TBS buffer containing 15% glycerol and dounce homogenized, followed by solubilization in 40 mM dodecyl-β-d-maltopyranoside (DDM or C12M) and 8 mM cholesterol hemisuccinate tris salt (CHS). Isolation of the soluble fraction was performed by ultracentrifugation, and the supernatant was then incubated with TALON resin in the presence of 10 mM imidazole at 4°C for 1 to 2 hours. An XK-16 column is then packed with sample-laden resin and washed with two column volumes of buffer (TBS plus 5% glycerol, 1 mM C12M, and 0.2 mM CHS at pH 8.0) containing 20 mM imidazole and 10 column volumes containing 30 mM imidazole and eluted with buffer containing 250 mM imidazole. Protein-containing fractions were then concentrated and digested with HRV 3C protease (1:25, w/w) at 4°C overnight. The digested protein was then ultracentrifuged and injected onto a Superdex 200 10/300 GL column for size exclusion chromatography (SEC) using buffer equilibrated with 20 mM Hepes (pH 7.0), 100 mM NaCl, and 0.5 mM C12M. Fractional samples were analyzed by SDS-polyacrylamide gel electrophoresis and fluorescence SEC, pooled accordingly, and concentrated for cryo-EM grid preparation to ~5 mg/ml.

### Electron microscopy sample preparation

The preparation of rP2X_7_ complexes with different ligands was achieved by incubation of purified apo receptors with antagonists at three to four times excess concentrations of protomer, approximately 200 to 400 μM across all antagonists. Following a 1-hour incubation and ultracentrifugation (40,000 RPM, 1 hour), cryo-EM grids were prepared for each rP2X_7_ complex. All antagonist grids were created by applying the 2.5-μl sample to glow-discharged (15 mA, 1 min) Quantifoil R1.2/1.3 300 mesh gold holey carbon grids, which were blotted for 1.5 s under 100% humidity at 6°C. Immediately after application, grids were flash-frozen in liquid ethane using an FEI Vitrobot Mark IV and stored under liquid nitrogen until screening and large-scale data acquisition.

### Electron microscopy data acquisition

All Cryo-EM datasets for each receptor complex were collected on Titan Krios microscopes (FEI) operated at 300 kV at the Pacific Northwest Center for Cryo-EM. The acquisition of datasets was completed with an energy filter (Gatan Image Filter, 20-eV slit width) and a Gatan K3 direct-electron detector. Super-resolution mode was used to collect all movies at a nominal magnification of ×130,000, corresponding to a physical pixel size of ~0.648 Å/pixel, using a defocus range of −0.8 to −1.5 μm and a total dose of between 40 and 44 e^−^/Å^2^. In addition, each dataset used “multi-shot” and “multi-hole” collection schemes driven by serialEM to maximize high-throughput data collection (*59*).

### Electron microscopy data processing

Motion correction and binning of super-resolution image stacks were completed in cryoSPARC using patch motion correction (fig. S2 and table S1) (*60*). cryoSPARC was also used for the estimation of the contrast transfer function (CTF) parameters with patch CTF estimation, followed by particle picking using two-dimensional templates. Micrographs and particle picks were curated and after extraction, particles were classified using iterative ab initio and heterogeneous classifications (fig. S2 and table S1). Re-extraction of the homogenous particle stacks at the physical pixel size generated the final particle stacks, which were then subjected to CTF correction at the global and local scales (fig. S2 and table S1). A final nonuniform refinement generated the consensus cryo-EM map (fig. S2 and table S1).

### Model building and structure determination

The antagonist-bound models were built in Coot using the apo closed-state structure of rP2X_7_ as the initial model (Protein Data Bank code: 6U9V) (*16*, *61*). Ligands and their corresponding CIF files were built in eLBOW with protonation states corresponding to approximately pH 7 (*62*). All stages of model building involved manual adjustments based on the quality of the maps in Coot, followed by real-space refinement in PHENIX (*63*). Limited glycosylation and acylation were included in the models when justified by density. In some of the models, side chains for residues were not included if sufficient density was not apparent. Some heteroatoms were renumbered from the initial models to facilitate comparison between the final models. Model quality was evaluated by MolProbity (*64*).

### Two-electrode voltage clamping

#### Preparation of oocytes expressing P2XRs

Ecocyte Bioscience supplied defolliculated oocytes which were promptly resuspended in modified Barth’s solution containing the following: 88 mM NaCl, 1 mM KCl, 0.82 mM MgSO_4_, 0.33 mM Ca(NO_3_)_2_·4H_2_O, 0.41 mM CaCl_2_·2H_2_O, 2.4 mM NaHCO_3_, and 5 mM Hepes supplemented with amikacin 250 (mg/liter) and gentamycin (150 mg/liter). *Xenopus laevis* oocytes were then injected with either hP2X_1_ (50 nl of 100 ng/μl), hP2X_2_ (50 nl of 50 ng/μl), hP2X_3_ (50 nl of 400 ng/μl), hP2X_4_ (50 nl of 200 ng/μl), hP2X_7_ (50 nl of 10 ng/μl), or rP2X_7_ (50 nL of 20 ng/μl or 50 ng/μl) mRNA created from linearized full-length, wild-type or mutant pcDNA 3.1x according to the protocol provided in the mMESSAGE mMACHINE kit (Invitrogen). After injection, the oocytes were allowed to express protein for ~20 hours before recording was performed. Oocytes expressing hP2X_4_ were allowed to express protein for ~48 hours.

#### TEVC recording

All TEVC data were acquired with an Ooctye Clamp OC-725C amplifier, pClamp 8.2 software, and a gravity-fed RSC-200 Rapid Solution Changer that flowed buffer at ~5 ml/min. For recording, Sutter filamented glass (10 cm in length with an inner diameter of 0.69 mm and an outer diameter of 1.2 mm) was used to impale oocytes and clamp the holding voltage at −60 mV. Recordings for hP2X_1_, hP2X_2_, hP2X_7_, and rP2X_7_ were performed in buffer with the following: 100 mM NaCl, 2.5 mM KCl, 0.1 mM EDTA, 0.1 mM flufenamic acid, and 5 mM Hepes at pH 7.4. Recordings for hP2X_3_ and hP2X_4_ were performed in buffer with the following: 10 mM Hepes (pH 7.4), 140 mM NaCl, 5 mM KCl, 2 mM CaCl_2_, 2 mM MgCl_2_, and 10 mM glucose. For TEVC experiments with P2X_7_, oocytes expressing hP2X_7_ or rP2X_7_ were facilitated with 100 μM ATP before recording the final data.

#### Inhibition dose-response (IC_50_) experiments

To evaluate the inhibitory response of an antagonist for a dilution series, an initial excitatory signal was evoked at a subtype-specific concentration of ATP. Next, the antagonist was applied at a test concentration for 60 s and then coapplied at the same test concentration of the antagonist with the identical subtype-specific concentration of ATP. The antagonized signal was then normalized against the preceding excitatory signal evoked by ATP for each individual oocyte tested. The pre- and post-antagonist signals were evoked by 10 μM ATP for hP2X_1_, 10 μM ATP for hP2X_2_, 1 μM ATP for hP2X_3_, 50 μM ATP for hP2X_4,_ and 100 μM ATP for P2X_7_. For the K297V mutation in rP2X_7_, pre- and post-antagonist signals were evoked by 300 μM ATP. Each dataset was then fit to a nonlinear regression named “[inhibitor] vs. response – Variable slope (four parameters)” in GraphPad Prism 9 to produce a sigmoidal curve and afford an IC_50_ value. Each singular condition was used to generate an average which is reported plus or minus its SD between three discrete trials.

#### Recovery of current after complete inhibition (IC_100_) experiments

After testing an oocyte with a brief 100 μM ATP excitatory current, an antagonist is applied for 40, 120, or 240 s at IC_100_ calculated from IC_50_ values. The oocyte is then treated with 20 s of antagonist at the IC_100_ plus 100 μM ATP to see complete antagonism. This is followed by 120 s of washing with buffer, ending with a 20-s reapplication of 100 μM ATP. The signal after the reapplication of ATP was then compared to the initial excitatory current to afford a percentage of recovery of activation after complete antagonism. Experiments were run in triplicate. Trials were averaged and errors were reported as SD. The recovery of the receptor was tested after the application of an IC_100_ of each drug at the following concentrations: A438079 at 6.6 μM, A839977 at 1 μM, AZD9056 at 3 μM, GSK1482160 at 81 μM, methyl blue at 108 μM, and JNJ47965567 at 1.3 μM.

### In vitro biotinylation of rP2X_7_

Using a HiTrap desalting column (Cytiva), 1 ml of diluted purified rP2X_7_ was desalted into PBS buffer (pH 7.4). Protein-containing fractions were combined and concentrated before adjusting the pH to 8.0 with NaHCO_3_. NHS-PEG12-Biotin (Thermo Fisher Scientific) was added and incubated at 23°C for 60 min while shaking. PBS was used to dilute the biotinylated sample before four cycles of dilution and concentration to ensure the removal of free NHS-PEG12-Biotin via a 100-kDa concentrator (Millipore Sigma). After the final cycle, the absorbance at 280 nm of the concentrated sample was taken to quantify the amount of biotinylated rP2X_7_.

### BLI for affinity determination

A ForteBio Octet RED384 instrument with ForteBio Data Acquisition software 11.0 was used to carry out BLI experiments. The assays were performed at 30°C using 384-well tilted-bottom plates (Sartorius) while orbital shaking at 1000 RPM. Running buffer consisting of filtered (0.22 μm) PBS (pH 7.4) with 0.5 mM DDM (Anatrace). Streptavidin (SA) biosensor tips (Sartorius) were pre-equilibrated in running buffer for 20 min and loaded with 50 μg ml^−1^ of biotinylated rP2X_7_ ligand or biocytin (Sigma-Aldrich) control for 1800s. Blocking of the loaded SA sensors using 100 μg ml^−1^ of biocytin was then initiated for 150 s before washing with a running buffer for 60 s. A baseline of loaded sensors was taken in the running buffer for 120 s before dipping them into wells containing ligand dilutions (0.37 to 10 μM for methyl blue) for 600 s, and then returning the sensors to the running buffer for a 600-s disassociation step. Rate constants for association and disassociation were determined using ForteBio Data Analysis HT 11.0 evaluation software. The raw data were double reference subtracted for maximal signal between the large ligand and small analyte. Biocytin controls were the first subtraction across analyte concentrations, and the zero-analyte concentration was the second. The association rate constant (*k*_a_) and the dissociation rate constant (*k*_d_) were calculated for each interaction pair using an average of three independent assays containing at least four different concentrations that were globally fit to a 2:1 Langmuir binding model. Equilibrium dissociation constants (*K*_D_) were calculated as the ratio of *k*_d_ to *k*_a_. The *K*_D1_, *K*_D2_, *k*_a1_, *k*_a2_, *k*_d1_, and *k*_d2_ values are reported as the average ± the SD of three replicate datasets for each analyte, with each dataset using at least four independent traces. The analyzed data were exported and plotted in GraphPad Prism 9.0.

## Supplementary Materials

### This PDF file includes:

Figs. S1 to S10 Tables S1 to S3 References
