# Supplementary material for: P2X7 receptors exhibit at least three modes of allosteric antagonism
Source: Sci Adv. 2024 Oct 4;10(40):eado5084. doi: 10.1126/sciadv.ado5084 (PMC11451537; doi:10.1126/sciadv.ado5084)
Supplement: Supplementary file 1 — Figs. S1 to S10 Tables S1 to S3 References [file sciadv.ado5084_sm.pdf]

Science Advances
AAAS

Supplementary Materials for

$P2X_7$ receptors exhibit at least three modes of allosteric antagonism

Adam C. Oken *et al.*

Corresponding author: Steven E. Mansoor, mansoors@ohsu.edu

Sci. Adv. **10**, eado5084 (2024)
DOI: 10.1126/sciadv.ado5084

This PDF file includes:

Figs. S1 to S10
Tables S1 to S3
References

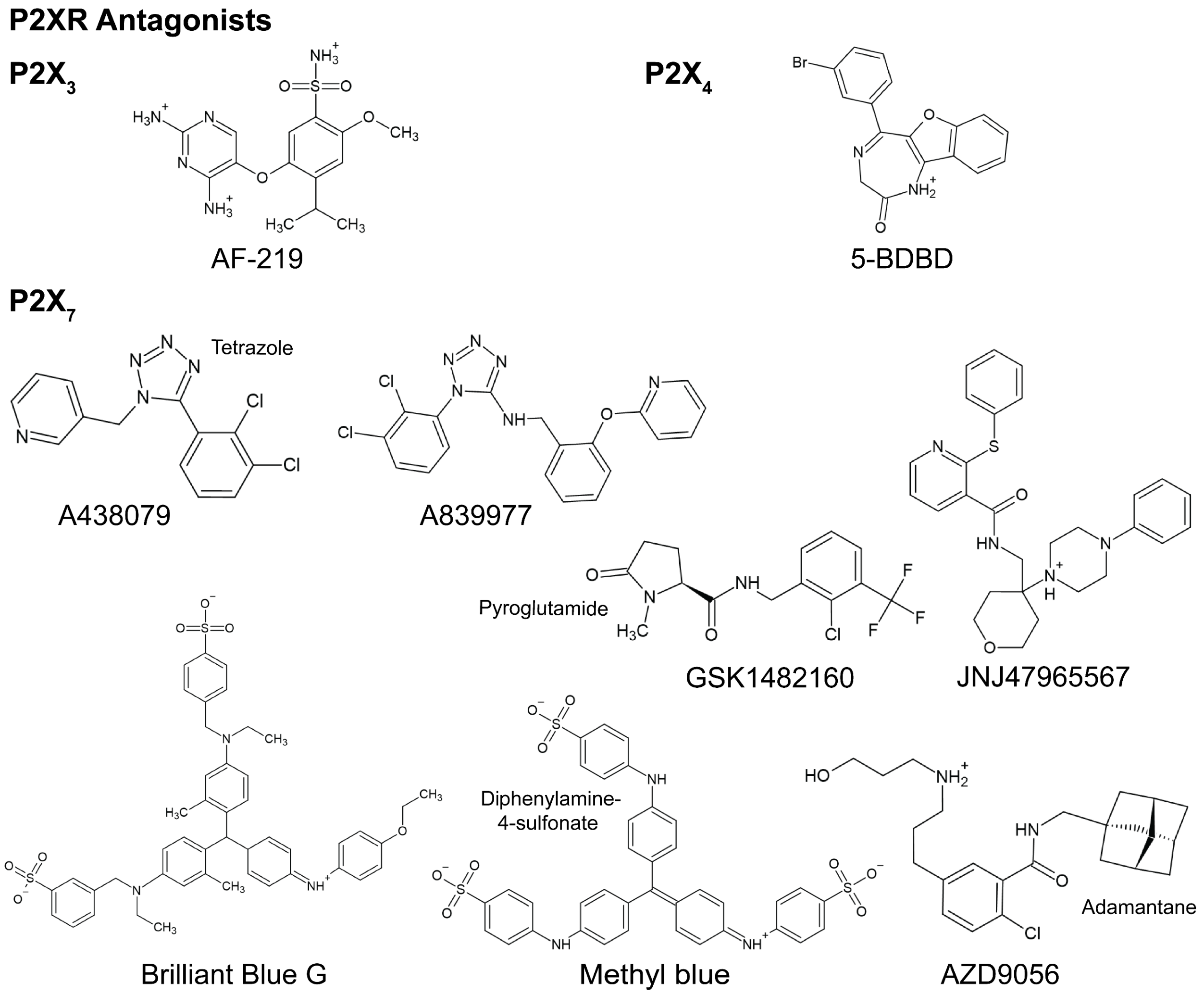

Fig. S1. Chemical structures and functional group names of P2XR antagonists.

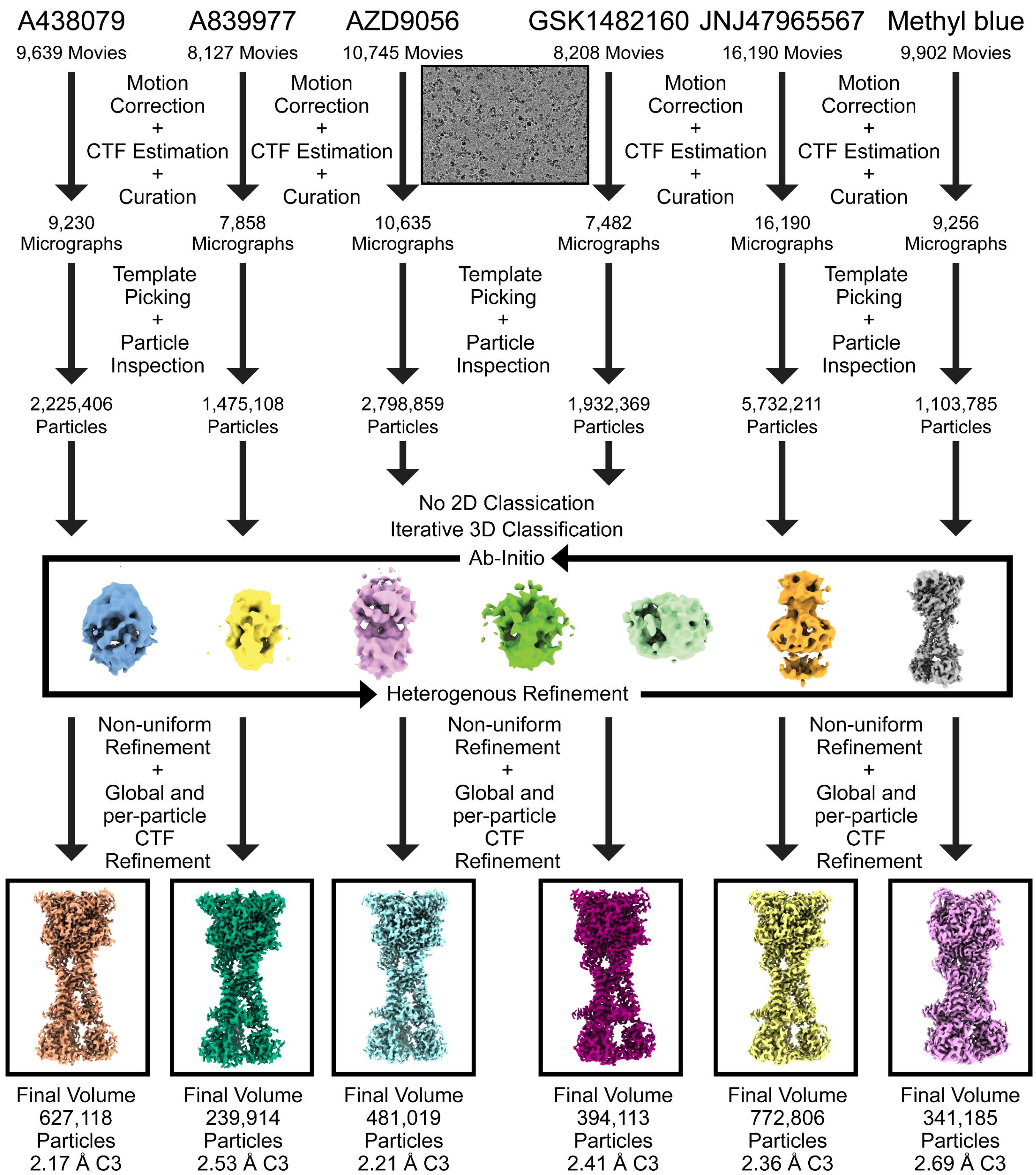

Fig. S2. General cryo-EM processing pipeline for rP2X$_7$ reconstructions. After high-throughput data acquisition, movies are binned during patch motion correction, patch CTF correction performed, and usable micrographs selected in CryoSPARC (*60*). Accepted micrographs were template picked using 2D templates generated from a low resolution rP2X$_7$ map, particles extracted, and sent directly to iterative 3D classification using ab-initio and heterogenous classification jobs in CryoSPARC. No 2D classification was performed for any dataset. After a final particle stack is determined, particles are re-extracted at the full pixel size, CTF corrected at both the global and per-particle levels, and refined using CryoSPARC's non-uniform refinement. The AZD9056 and methyl blue datasets were processed with C1 symmetry

and showed nearly identical density for ligands and sidechains of residues within the classical and extended allosteric ligand-binding sites. The pore architecture in maps refined with C1 symmetry were also nearly identical to maps refined with C3 symmetry (Fig. S4B).

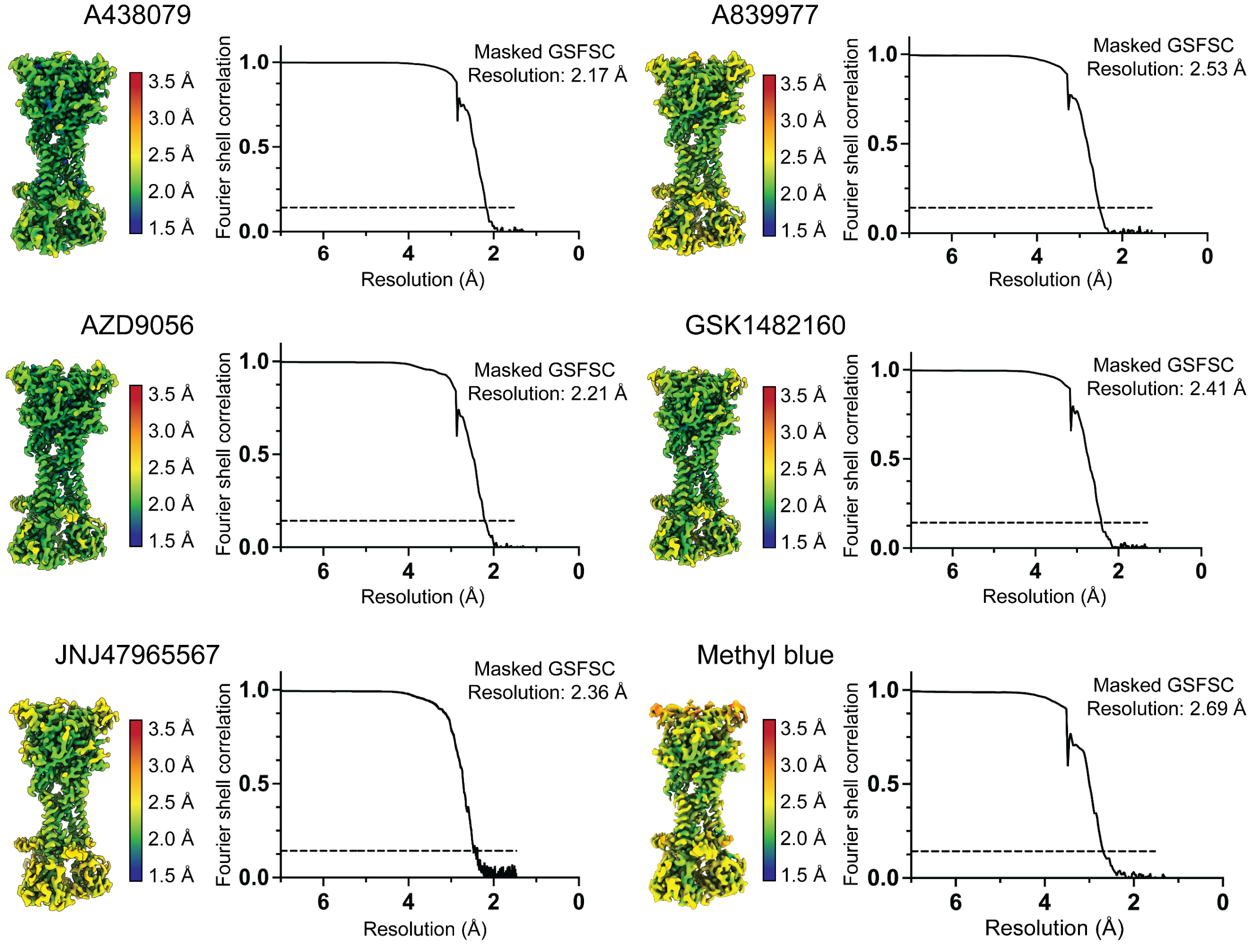

Fig. S3. Local resolution and Fourier shell correlation (FSC) plots for all rP2X$_7$ datasets. Resolution stated is at an FSC=0.143. All local resolution plots range between 1.5 Å (blue) and 3.5 Å (red).

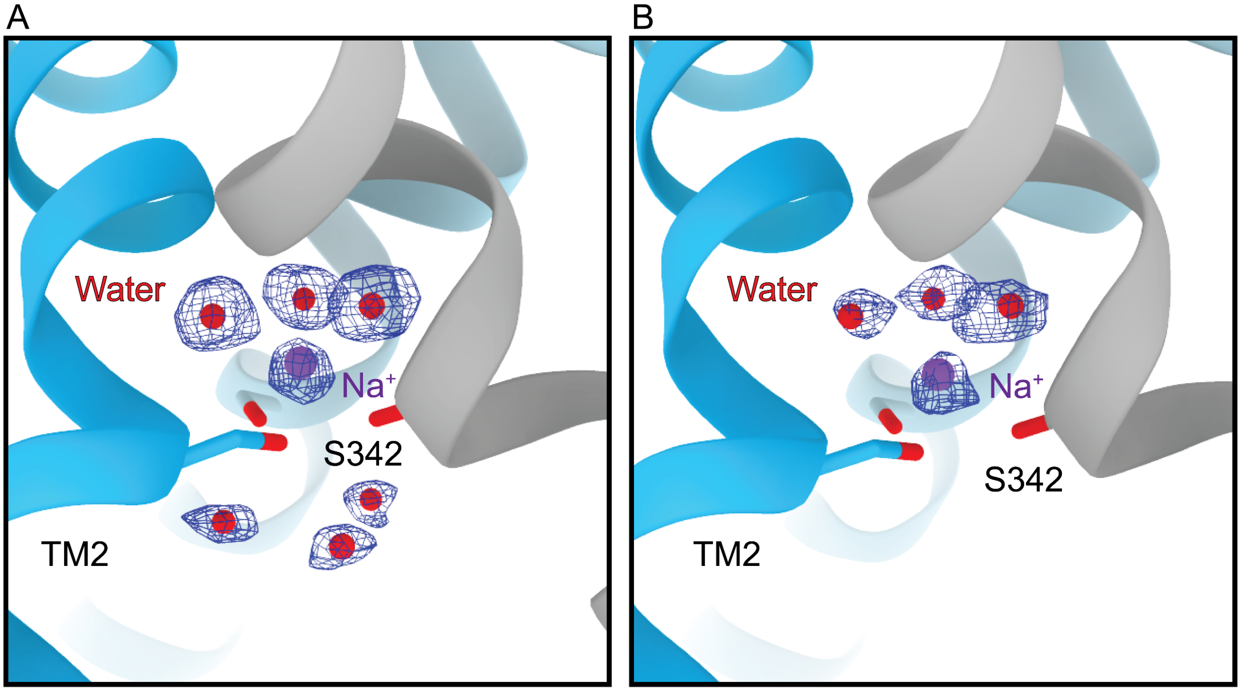

Fig. S4. Sodium coordination directly above the gate in antagonist-bound inhibited state structures. (**A**) Sodium coordination in the 2.2 Å A438079-bound $rP2X_7$ structure with its C3 refined cryo-EM density shown in blue mesh. The Na^+ ion (purple sphere), found directly above the gate (formed by TM2 from each protomer), is partially hydrated, coordinated in octahedral geometry by three water molecules (red spheres) and S342 from each protomer **(***48***)**. Each water below the gate forms a hydrogen bond with its neighboring S342 (distance of ~2.7 Å). Our data provides no insights into the permeability or dehydration process of other cations (such as K^+ or Ca^{2+}) in the pore **(***66-68***)**. Our vitrification buffer only contained Na^+ ions. (**B**) Sodium coordination in a C1 refined cryo-EM map of AZD9056 bound to $rP2X_7$ at 2.5 Å resolution with its corresponding electron density shown in blue mesh. The partially hydrated Na^+ ion is still present without symmetry imposed during data processing (C1), coordinated in octahedral geometry by three water molecules (red spheres) and S342 from each protomer. The AZD9056-bound $rP2X_7$ dataset was used for C1 refinement to allow visualization of features at a sufficient resolution to readily distinguish the Na^+ ion and waters.

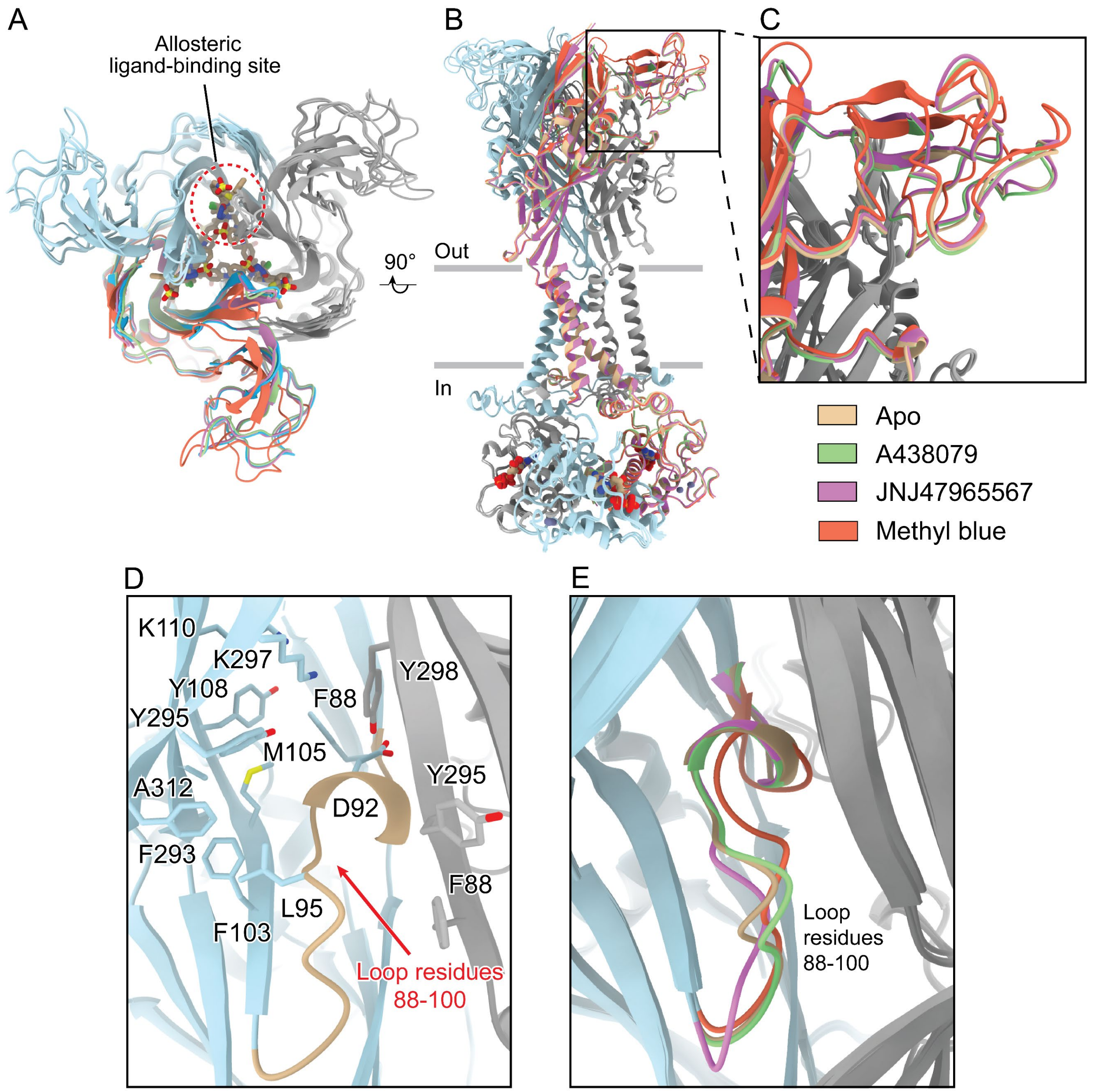

Fig. S5. Comparison of structural changes between apo closed (tan), A438079- (light green), JNJ47965567- (light purple), and methyl blue- (light red) bound rP2X$_7$ structures. (**A**) Top-down view of the receptor highlighting the movements of loops in the head domain between apo closed and representative antagonist-bound inhibited state structures of rP2X$_7$ (PDB code: 8TR5) (*48*). One protomer is uniquely colored by the respective structure with the apo structure in tan, the A438079-bound structure in light green, the JNJ47965567-bound structure in light purple, and the methyl blue-bound structure in light red. The other two protomers are colored light blue and gray. (**B**) Full receptor view highlighting that there are no significant changes to the transmembrane domains or N- and C-termini between apo closed and representative antagonist-bound inhibited state structures of rP2X$_7$ (PDB code: 8TR5) (*48*). Compared to the apo closed state structure, the mean RMSD at Cα carbons for all ligand-bound structures at residues 6-48

and 330-595 (the receptor excluding the extracellular domain) is only 0.5 Å. (**C**) Magnified view of panel B highlighting the small, but significant structural movements between the extracellular loops across the four representative structures of rP2X$_7$. Compared to the apo closed state structure, the RMSD's at Cα carbons of the extracellular domain (residues 49-329) for the A438079-bound, JNJ47965567-bound, and methyl blue-bound inhibited state structures are 0.48 Å, 0.70 Å, and 2.3 Å, respectively. (**D**) Ribbon representation of the classical allosteric ligand-binding site in the apo closed state rP2X$_7$ structure (PDB code: 8TR5) (*48*). Residues known to coordinate ligands and loop residues displaced by allosteric antagonists are labeled (colored in tan to represent their positions in the apo closed state). (**E**) View of a loop in the upper body domain (residues 88-100 of rP2X$_7$) that is displaced to accommodate allosteric antagonists in the classical allosteric ligand-binding site, allowing for additional ligand-receptor interactions. The RMSD of Cα carbon positions for these thirteen residues of the loop between the apo closed state structure and the A438079-bound, JNJ47965567-bound, and methyl blue-bound structures are 0.65 Å, 2.4 Å, 2.7 Å, respectively. This mobile loop is uniquely displaced when methyl blue binds compared to A438079-bound and JNJ47965567-bound conformational states, as evidenced by a RMSD at Cα carbons of 2.7 and 3.8 Å, respectively.

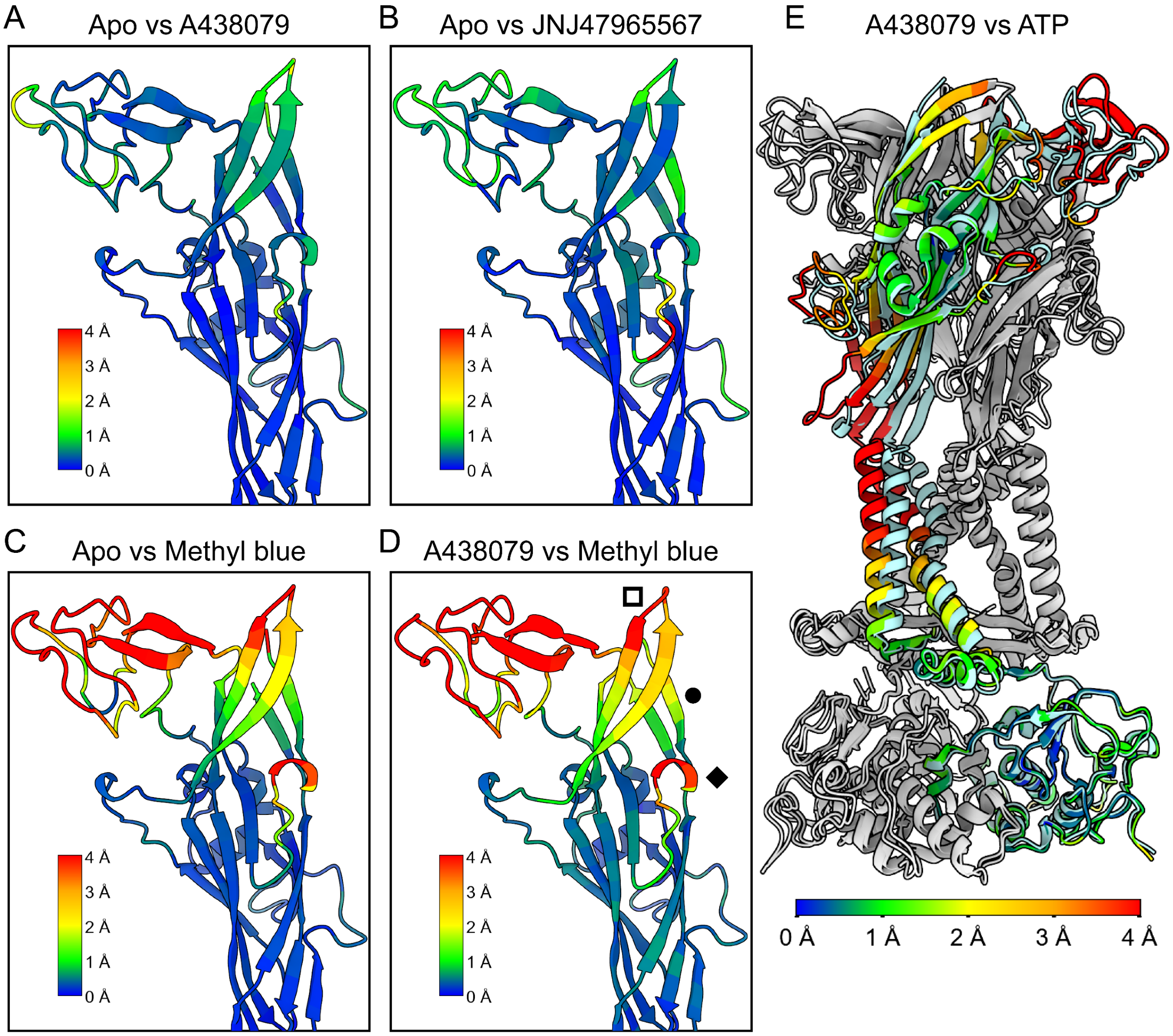

Fig. S6. Differences between apo closed, ATP-bound open, and representative antagonist-bound inhibited state structures of rP2X$_7$ rendered by RMSD as calculated in ChimeraX (*69*)**.** (**A-C**) The extracellular domain of one rP2X$_7$ protomer in the apo closed state structure compared to the A438079-bound (A), JNJ47965567-bound (B), or methyl blue-bound (C) inhibited state structures highlighting differences in domains colored by RMSD mapped onto the apo closed structure (PDB code: 8TR5) (*48*). The A438079-bound structure is similar, while the methyl blue-bound structure differs from the apo closed state structure. (**D**) The extracellular domain of one rP2X$_7$ protomer in the A438079-bound structure compared to the methyl blue-bound structure, highlighting stark differences in the extracellular domain that occur to accommodate the larger ligand. The symbol ◆ marks the location of loop 88-100, the symbol ● represents loop 70-85, and the symbol □ represents loop 296-308. (**E**) Full receptor view of the A438079-bound inhibited state structure (gray and light blue) aligned with the ATP-bound open state structure (gray and colored, PDB code: 6U9W) of rP2X$_7$ (*16*). One protomer of the ATP-bound open state structure is colored by RMSD to highlight the movements between the two distinct conformational states.

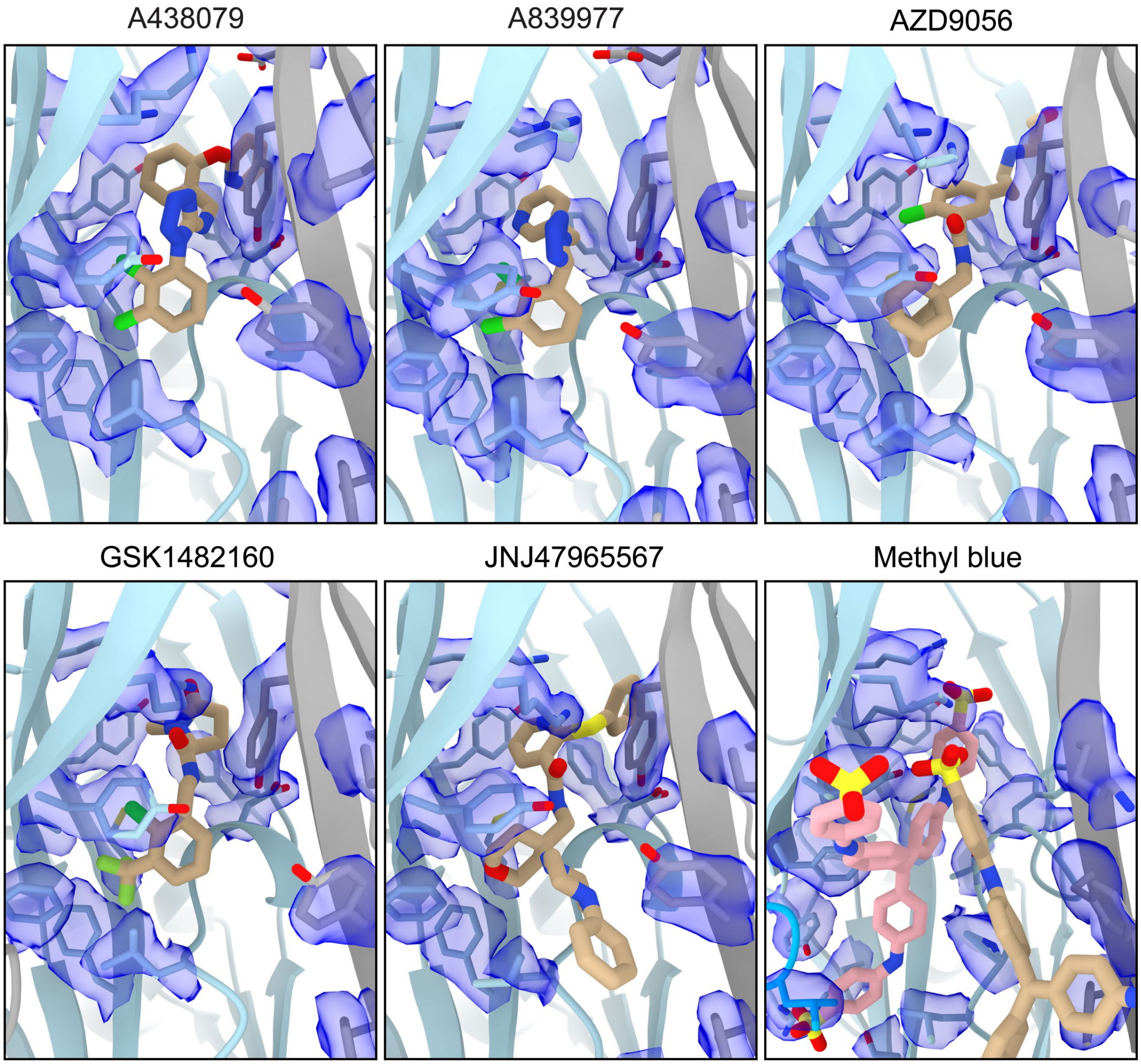

Fig. S7. Side-chain density of residues within the allosteric ligand-binding sites for each of the six antagonists. Side chain densities for key residues within allosteric pockets shown in transparent blue.

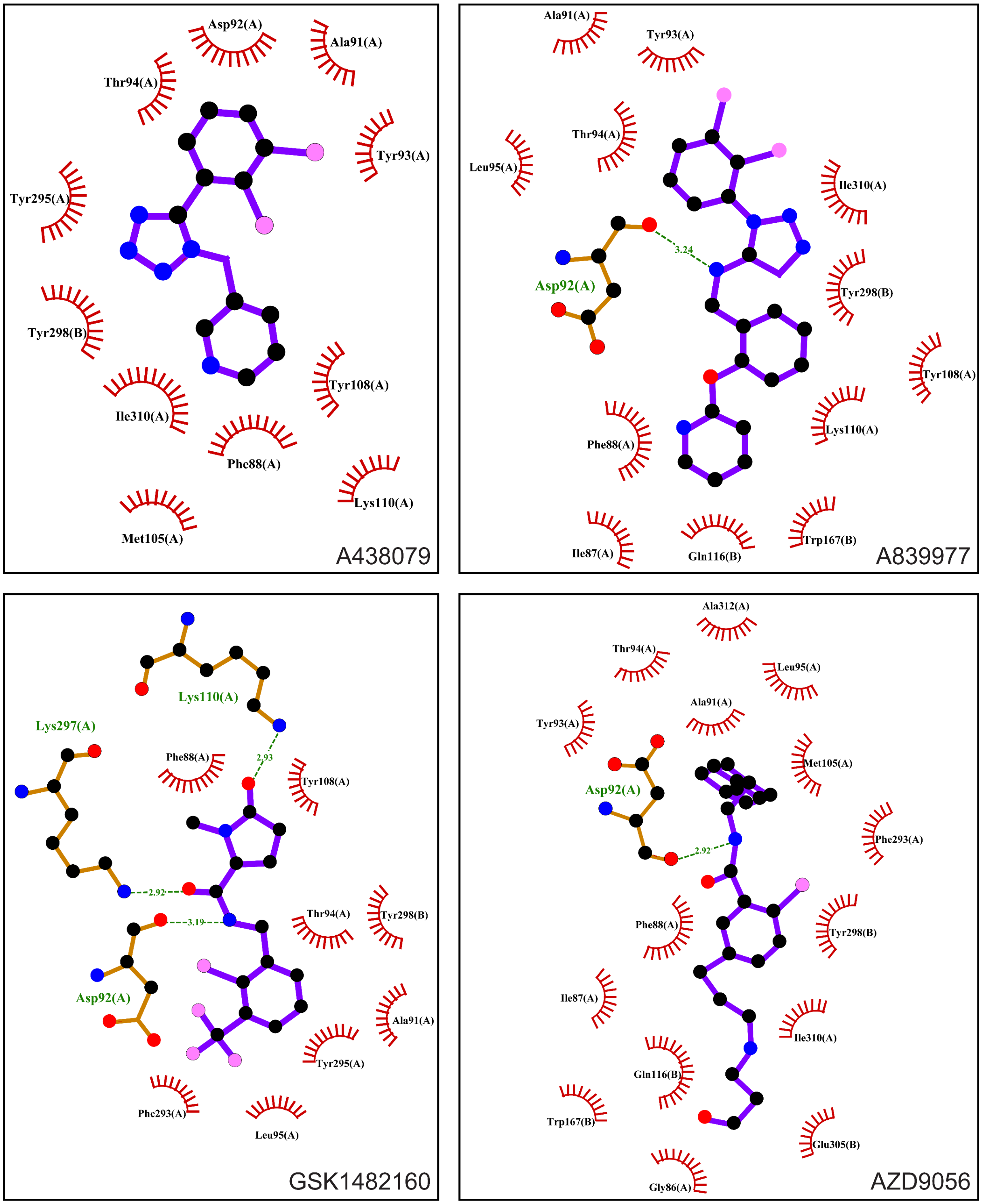

Fig. S8. LigPlot diagrams for the shallow binding allosteric antagonists (*70*). These diagrams highlight the interactions between $rP2X_7$ and the shallow ligands A438079, A839977, GSK1482160, and AZD9056. In these LigPlot diagrams, A839977, GSK1482160, and AZD9056 form interactions with D92. In addition, K297 and K110 form interactions with GSK1482160.

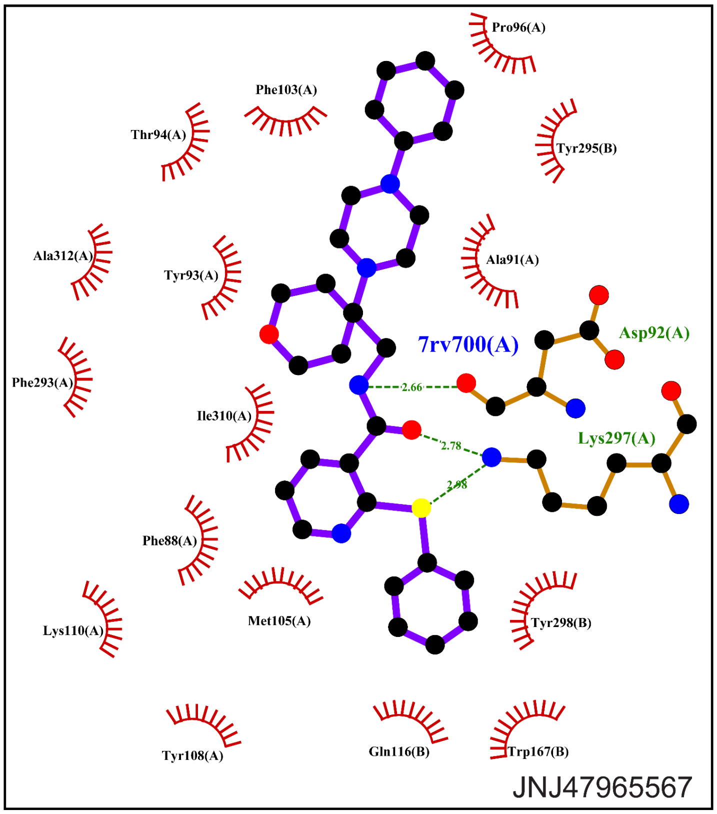
Pro96(A)
Phe103(A)
Thr94(A)
Tyr295(B)
Ala312(A)
Tyr93(A)
Ala91(A)
7rv700(A)
Asp92(A)
Phe293(A)
2.66
Ile310(A)
2.78
Lys297(A)
2.98
Phe88(A)
Met105(A)
Tyr298(B)
Lys110(A)
Tyr108(A)
Gln116(B)
Trp167(B)
JNJ47965567

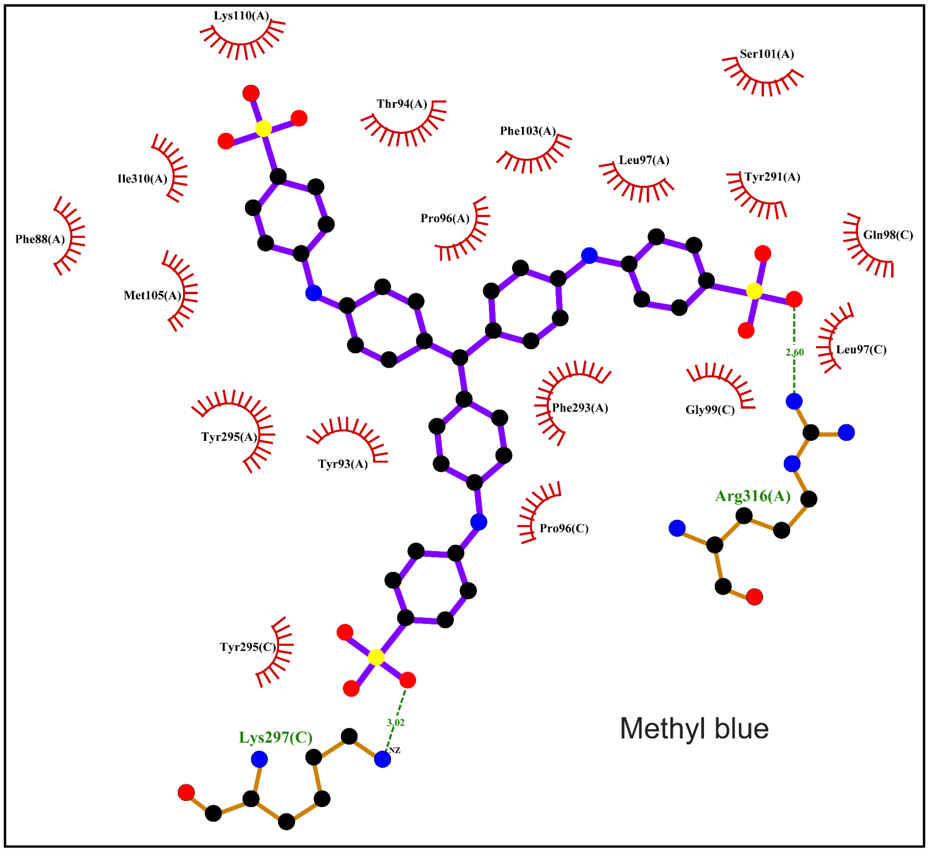
Lys110(A)
Ser101(A)
Thr94(A)
Phe103(A)
Leu97(A)
Ile310(A)
Tyr291(A)
Pro96(A)
Gln98(C)
Phe88(A)
Met105(A)
2.60
Leu97(C)
Phe293(A)
Gly99(C)
Tyr295(A)
Tyr93(A)
Arg316(A)
Pro96(C)
Tyr295(C)
3.02
NZ
Lys297(C)
Methyl blue

Fig. S9. LigPlot diagrams for the deep and starfish binding allosteric antagonists (*70*). These diagrams highlight the interactions between $rP2X_7$ and the deep binder JNJ47965567 as well as $rP2X_7$ and the starfish binder methyl blue. JNJ47965567 forms interactions with D92 and K297 while methyl blue forms interactions with K297 and R316.

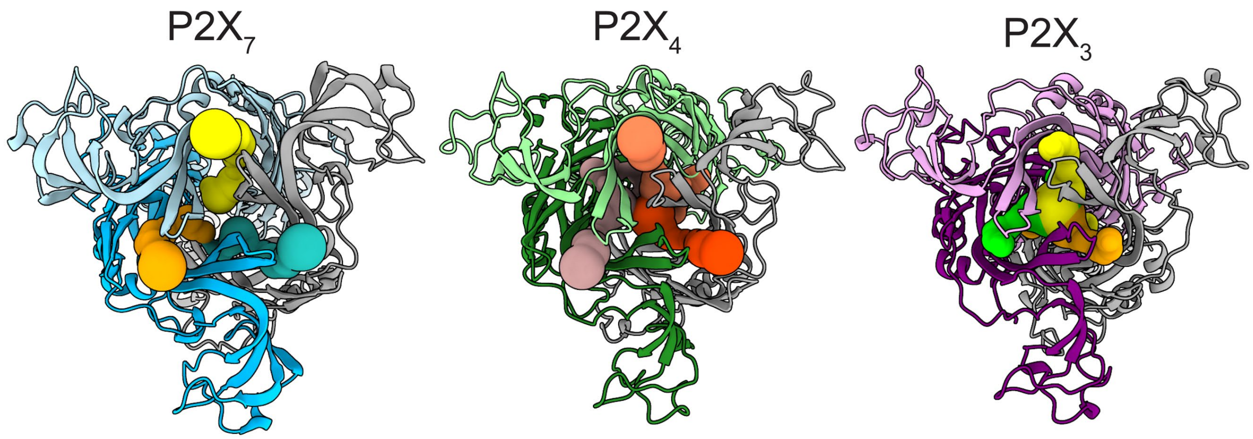

Fig. S10. Surface accessible area of allosteric sites across P2XR subtypes with known structure. Surface accessible area was calculated using the default settings on the Mole online server (*71,72*). Across P2XRs, the location of the classical allosteric ligand-binding site in the apo closed state of rP2X_7 (blue, light blue, and gray) becomes progressively smaller in the same location of the apo closed states of zfP2X_4 (green, light green, and gray) and hP2X_3 (purple, pink, and gray), respectively. The pockets in each receptor are colored for visual purposes only.

Table S1. Cryo-EM collection, refinement, and validation statistics.

	A438079 rP2X$_7$ (EMD-41571) (PDB: 8TR6)	A839977 rP2X$_7$ (EMD-41572) (PDB: 8TR7)	AZD9056 rP2X$_7$ (EMD-41573) (PDB: 8TR8)	GSK1482160 rP2X$_7$ (EMDB-41575) (PDB: 8TRA)	JNJ47965567 rP2X$_7$ (EMD-41576) (PDB: 8TRB)	Methyl blue rP2X$_7$ (EMD-41582) (PDB: 8TRK)
**Data collection and processing**						
Magnification (kx)	130	130	130	130	130	130
Voltage (kV)	300	300	300	300	300	300
Electron exposure (e–/Å^2)	42	43	44	44	42	42
Movie frames	42	48	64	50	42	50
Defocus range (μm)	-0.8 to -1.4	-0.9 to -1.4	-1.0 to -1.7	-1.0 to -1.7	-0.8 to -1.4	-0.9 to -1.5
Pixel size (Å)	0.648 (0.324 super-res)	0.648 (0.324 super-res)	0.648 (0.324 super-res)	0.648 (0.324 super-res)	0.648 (0.324 super-res)	0.648 (0.324 super-res)
Symmetry imposed	C3	C3	C3	C3	C3	C3
Initial micrographs (no.)	9,639	8,127	10,745	8,208	16,190	9,902
Final micrographs used (no.)	9,230	7,858	10,635	7,482	16,190	9,256
Initial particle images (no.)	2,225,406	1,475,108	2,798,859	1,932,369	5,732,211	1,103,785
Final particle images (no.)	627,118	239,914	481,019	394,113	772,806	341,185
Map resolution (Å) FSC threshold	2.17 (0.143)	2.53 (0.143)	2.21 (0.143)	2.41 (0.143)	2.36 (0.143)	2.69 (0.143)
Map resolution range (Å)	1.4 to 22	1.4 to 7.6	1.4 to 8.1	1.4 to 9.5	1.4 to 11	1.6 to 8.7
**Refinement**						
Initial model used (PDB code)	6U9V	6U9V	6U9V	6U9V	6U9V	6U9V
Model resolution (Å) FSC threshold	2.15 (0.143)	2.50 (0.143)	2.20 (0.143)	2.38 (0.143)	2.33 (0.143)	2.66 (0.143)
Map sharpening *B* factor (Å^2)	67.1	81.4	65.3	80	100.4	95
**Model composition**						
Non-hydrogen atoms	14,050	14,032	14,006	13,937	14,080	13,849
Protein Residues	1662	1662	1653	1662	1659	1656
Ligands	25	22	22	22	22	30
Waters	276	290	352	196	312	85
B factors (Å^2)						
Protein	0.63/77.1/27.3	17.0/82.1/44.5	6.0/63.8/31.4	6.5/55.6/ 27.5	1.11/70.9/28.3	3.1/109/ 36.4
Ligand	5.56/75.5/24.0	25.1/87.4/35.2	14.2/72.6/24.1	17.8/69.2/25.5	4.10/73.5/18.1	22.6/59.3/39.8
Nucleotide	42.1/42.1/42.1	67.4/67.4/67.4	47.3/47.3/47.3	35.8/35.8/35.8	52.5/52.5/52.5	29.0/29.0/29.0
Water	1.62/30.2/13.9	14.9/69.9/26.0	5.5/46.8/14.7	3.92/30/ 22.6	0.1/40.3/ 8.8	2.7/33.2/ 17.9
R.m.s. deviations						
Bond lengths (Å)	0.004 (0)	0.008 (0)	0.006 (0)	0.003 (0)	0.007 (0)	0.009 (0)
Bond angles (°)	0.689 (0)	0.661 (0)	0.666 (0)	0.619 (0)	0.700 (0)	0.803 (0)
Validation						
MolProbity score	1.31	1.17	1.08	1.1	1.14	1.23
Clash score	5.51	2.37	2.13	2.63	2.36	2.91
Poor rotamers (%)	0.62	0	0	0.21	0.00	0.00
Ramachandran plot						
Favored (%)	97.95	97.14	97.57	97.77	97.39	97.2
Allowed (%)	2.05	2.86	2.43	2.23	2.61	2.8
Disallowed (%)	0	0	0	0	0	0

Table S2. Effects of mutations to the allosteric ligand-binding sites of rP2X$_7$.

	A43807	A839977	AZD9056	GSK148216	JNJ479655	Methyl blue
WT	550 ± 113 nM	116 ± 13 nM	300 ± 100 nM	3.6 ± 0.5 µM	22 ± 6 nM	4 ± 1 µM
F88A	14 ± 1 µM	1.9 ± 0.2 µM	130 ± 10 nM	13 ± 3 µM	0.8 ± 0.1 µM	5 ± 2 µM
F103A	>10 µM	>1 µM	>10 µM	>20 µM	50-80% inhibition at 625 nM	50-75% inhibition at 30 µM
K297V	110 ± 90 nM	3.2 ± 0.6 nM	12 ± 1 nM	1.3 ± 0.4 µM	21 ± 6 nM	4 ± 1 µM

Mutation of residues within the classical allosteric pocket, which exists at the interface between adjacent protomers, resulted in non-functional receptors. However, the effects of three select mutations were quantified by TEVC. F88 is a hydrophobic residue located at the entrance of the classical allosteric pocket and has been shown to impact ligand potency (*47*). Mutation of F88 to an alanine residue generally resulted in decreased inhibitory potencies, consistent with previous data of other P2X$_7$ antagonists (*47,50,51*). F103 is a hydrophobic residue located deep within the classical allosteric pocket. While expression of the F103A receptor in oocytes was atypical (reduced expression and variable response to ATP), the mutation generally decreased ligand potency. Finally, K297 is a residue located in the middle of the classical allosteric pocket and forms hydrogen bonding interactions with some ligands. Mutation of K297 to a valine residue increases or does not affect the inhibitory potency of the tested ligands. While the K297V mutation removes a hydrogen bonding interaction, there are likely additional hydrophobic interactions that compensate. Attempted mutations that produced non-functional channels by TEVC include: rP2X$_7$-K297A, rP2X$_7$-K297G, rP2X$_7$-K297M, rP2X$_7$-K297Q, rP2X$_7$-Y298A, rP2X$_7$-Y298G, rP2X$_7$-Y298V, rP2X$_7$-Y298L, rP2X$_7$-R316A, rP2X$_7$-R316G, rP2X$_7$-R316Q, rP2X$_7$-R316H, and rP2X$_7$-Y295A. P2X$_7$ receptors with mutations were considered non-functional if, after 24 or 48 hours of expression, no current was observed after repeated application of 100 µM ATP.

Table S3. Recovery of activation values following an IC_{100} of antagonist at $rP2X_7$

	A438079 (%)	A839977 (%)	AZD9056 (%)	GSK1482160 (%)	JNJ47965567 (%)	Methyl blue (%)
40 seconds	81 ± 5	69 ± 6	82 ± 5	69 ± 4	1.4 ± 0.2	53 ± 4
120 seconds	48 ± 15	6.7 ± 2	47 ± 9	45 ± 6	1.6 ± 1	15 ± 5
240 seconds	50 ± 11	1.4 ± 0.3	29 ± 15	29 ± 9	2 ± 1	4 ± 1

Recovery of $P2X_7$ receptor activation expressed as a percentage of baseline current in response to 100 μM ATP following variable IC_{100} application times of antagonist (40 s, 120 s, and 240 s) and a fixed buffer wash (120 s).

REFERENCES AND NOTES

1. A. Surprenant, F. Rassendren, E. Kawashima, R. A. North, G. Buell, The cytolytic P2Z receptor for extracellular ATP identified as a P2X receptor (P2X7). *Science* **272**, 735–738 (1996).

2. S. K. Baljit, B. Geoffrey, K. Charles, F. K. Brian, R. A. North, S. Philippe, V. Mark, P. A. H. Patrick, International union of pharmacology. XXIV. Current status of the nomenclature and properties of P2X receptors and their subunits. *Pharmacol. Rev.* **53**, 107–118 (2001).

3. R. A. North, Molecular physiology of P2X receptors. *Physiol. Rev.* **82**, 1013–1067 (2002).

4. S. Valera, N. Hussy, R. J. Evans, N. Adami, R. A. North, A. Surprenant, G. Buell, A new class of ligand-gated ion channel defined by P2x receptor for extracellular ATP. *Nature* **371**, 516–519 (1994).

5. A. J. Brake, M. J. Wagenbach, D. Julius, New structural motif for ligand-gated ion channels defined by an ionotropic ATP receptor. *Nature* **371**, 519–523 (1994).

6. G. Burnstock, C. Kennedy, P2X receptors in health and disease. *Adv. Pharmacol.* **61**, 333–372 (2011).

7. G. Burnstock, Purinergic nerves. *Pharmacol. Rev.* **24**, 509–581 (1972).

8. A. L. Taylor, L. M. Schwiebert, J. J. Smith, C. King, J. R. Jones, E. J. Sorscher, E. M. Schwiebert, Epithelial P2X purinergic receptor channel expression and function. *J. Clin. Invest.* **104**, 875–884 (1999).

9. R. D. Murrell-Lagnado, O. S. Qureshi, Assembly and trafficking of P2X purinergic receptors (Review). *Mol. Membr. Biol.* **25**, 321–331 (2008).

10. A. Saul, R. Hausmann, A. Kless, A. Nicke, Heteromeric assembly of P2X subunits. *Front. Cell. Neurosci.* **7**, 250 (2013).

11. A. C. Oken, I. Krishnamurthy, J. C. Savage, N. E. Lisi, M. H. Godsey, S. E. Mansoor, Molecular pharmacology of P2X receptors: Exploring druggable domains revealed by structural biology. *Front. Pharmacol.* **13**, 925880 (2022).

12. S. E. Mansoor, How structural biology has directly impacted our understanding of P2X receptor function and gating, in *The P2X7 Receptor:Methods and Protocols*, A. Nicke, Ed., Methods in Molecular Biology (Humana Press, ed. 1, 2022), pp. 1–29. In press.

13. P. Werner, E. P. Seward, G. N. Buell, R. A. North, Domains of P2X receptors involved in desensitization. *Proc. Natl. Acad. Sci. U.S.A.* **93**, 15485–15490 (1996).

14. T. Kawate, P2X receptor activation. *Adv. Exp. Med. Biol.* **1051**, 55–69 (2017).

15. S. E. Mansoor, W. Lu, W. Oosterheert, M. Shekhar, E. Tajkhorshid, E. Gouaux, X-ray structures define human $P2X_3$ receptor gating cycle and antagonist action. *Nature* **538**, 66–71 (2016).

16. A. E. McCarthy, C. Yoshioka, S. E. Mansoor, Full-length $P2X_7$ structures reveal how palmitoylation prevents channel desensitization. *Cell* **179**, 659–670.e13 (2019).

17. J. Kamei, Y. Takahashi, Y. Yoshikawa, A. Saitoh, Involvement of P2X receptor subtypes in ATP-induced enhancement of the cough reflex sensitivity. *Eur. J. Pharmacol.* **528**, 158–161 (2005).

18. C. Furlan-Freguia, P. Marchese, A. Gruber, Z. M. Ruggeri, W. Ruf, P2X7 receptor signaling contributes to tissue factor-dependent thrombosis in mice. *J. Clin. Invest.* **121**, 2932–2944 (2011).

19. M. P. Mahaut-Smith, S. Jones, R. J. Evans, The P2X1 receptor and platelet function. *Purinergic Signal* **7**, 341–356 (2011).

20. R. A. North, M. F. Jarvis, P2X receptors as drug targets. *Mol. Pharmacol.* **83**, 759–769 (2013).

21. D. Yan, Y. Zhu, T. Walsh, D. Xie, H. Yuan, A. Sirmaci, T. Fujikawa, A. C. Y. Wong, T. L. Loh, L. Du, M. Grati, S. M. Vlajkovic, S. Blanton, A. F. Ryan, Z.-Y. Chen, P. R. Thorne, B. Kachar, M. Tekin, H.-B. Zhao, G. D. Housley, M.-C. King, X. Z. Liu, Mutation of the ATP-gated P2X2 receptor leads to progressive hearing loss and increased susceptibility to noise. *Proc. Natl. Acad. Sci. U.S.A.* **110**, 2228–2233 (2013).

22. G. Burnstock, G. E. Knight, The potential of P2X7 receptors as a therapeutic target, including inflammation and tumour progression. *Purinergic Signal* **14**, 1–18 (2018).

23. E. Martin, M. Amar, C. Dalle, I. Youssef, C. Boucher, C. Le Duigou, M. Brückner, A. Prigent, V. Sazdovitch, A. Halle, J. M. Kanellopoulos, B. Fontaine, B. Delatour, C. Delarasse, New role of P2X7 receptor in an Alzheimer's disease mouse model. *Mol. Psychiatry* **24**, 108–125 (2019).

24. L. Francistiova, C. Bianchi, C. Di Lauro, A. Sebastian-Serrano, L. de Diego-Garcia, J. Kobolak, A. Dinnyes, M. Diaz-Hernandez, The role of P2X7 receptor in Alzheimer's disease. *Front. Mol. Neurosci.* **13**, 94 (2020).

25. D. L. Donnelly-Roberts, M. F. Jarvis, Discovery of P2X7 receptor-selective antagonists offers new insights into P2X7 receptor function and indicates a role in chronic pain states. *Br. J. Pharmacol.* **151**, 571–579 (2007).

26. R. A. North, P2X3 receptors and peripheral pain mechanisms. *J. Physiol.* **554**, 301–308 (2004).

27. G. Burnstock, Introduction: P2 receptors. *Curr. Top. Med. Chem.* **4**, 793–803 (2004).

28. Z. Huang, N. Xie, P. Illes, F. Di Virgilio, H. Ulrich, A. Semyanov, A. Verkhratsky, B. Sperlagh, S.-G. Yu, C. Huang, Y. Tang, From purines to purinergic signalling: Molecular functions and human diseases. *Signal Transduct. Target. Ther.* **6**, 162 (2021).

29. Y. Zou, R. Yang, L. Li, X. Xu, S. A.-O. Liang, Purinergic signaling: A potential therapeutic target for depression and chronic pain. *Purinergic Signal* **19**, 163–172 (2023).

30. P. Illes, C. E. Muller, K. A. Jacobson, T. Grutter, A. Nicke, S. J. Fountain, C. Kennedy, G. Schmalzing, M. F. Jarvis, S. S. Stojilkovic, B. F. King, F. Di Virgilio, Update of P2X receptor

properties and their pharmacology: IUPHAR Review 30. *Br. J. Pharmacol.* **178**, 489–514 (2021).

31. F. Di Virgilio, The P2Z purinoceptor: An intriguing role in immunity, inflammation and cell death. *Immunol. Today* **16**, 524–528 (1995).

32. H. Kong, H. Zhao, T. Chen, Y. Song, Y. Cui, Targeted P2X7/NLRP3 signaling pathway against inflammation, apoptosis, and pyroptosis of retinal endothelial cells in diabetic retinopathy. *Cell Death Dis.* **13**, 336 (2022).

33. P. Pelegrin, P2X7 receptor and the NLRP3 inflammasome: Partners in crime. *Biochem. Pharmacol.* **187**, 114385 (2021).

34. J. Zhou, G. Tian, Y. Quan, J. Li, X. Wang, W. Wu, M. Li, X. Liu, Inhibition of P2X7 purinergic receptor ameliorates cardiac fibrosis by suppressing NLRP3/IL-1β pathway. *Oxid. Med. Cell. Longev.* **2020**, 7956274 (2020).

35. R. Kopp, A. Krautloher, A. Ramirez-Fernandez, A. Nicke, P2X7 interactions and signaling–making head or tail of it. *Front. Mol. Neurosci.* **12**, 183 (2019).

36. A. Franceschini, M. Capece, P. Chiozzi, S. Falzoni, J. M. Sanz, A. C. Sarti, M. Bonora, P. Pinton, F. Di Virgilio, The P2X7 receptor directly interacts with the NLRP3 inflammasome scaffold protein. *FASEB J.* **29**, 2450–2461 (2015).

37. Q. Wang, L. Wang, Y.-H. Feng, X. Li, R. Zeng, G. I. Gorodeski, P2X7 receptor-mediated apoptosis of human cervical epithelial cells. *Am. J. Physiol. Cell. Physiol.* **287**, C1349–C1358 (2004).

38. W. Wang, J. Xiao, M. Adachi, Z. Liu, J. Zhou, 4-Aminopyridine induces apoptosis of human acute myeloid leukemia cells via increasing [Ca2+]i through P2X7 receptor pathway. *Cell. Physiol. Biochem.* **28**, 199–208 (2011).

39. X. Chen, J. Hu, L. Jiang, S. Xu, B. Zheng, C. Wang, J. Zhang, X. Wei, L. Chang, Q. Wang, Brilliant Blue G improves cognition in an animal model of Alzheimer's disease and inhibits

amyloid-β-induced loss of filopodia and dendrite spines in hippocampal neurons. *Neuroscience* **279**, 94–101 (2014).

40. R. Lara, E. Adinolfi, C. A. Harwood, M. Philpott, J. A. Barden, F. Di Virgilio, S. McNulty, P2X7 in cancer: From molecular mechanisms to therapeutics. *Front. Pharmacol.* **11**, 793 (2020).

41. S. Roger, B. Jelassi, I. Couillin, P. Pelegrin, P. Besson, L.-H. Jiang, Understanding the roles of the P2X7 receptor in solid tumour progression and therapeutic perspectives. *Biochim. Biophys. Acta.* **1848**, 2584–2602 (2015).

42. B. G. Shokoples, P. Paradis, E. L. Schiffrin, P2X7 receptors: An untapped target for the management of cardiovascular disease. *Arterioscler. Thromb. Vasc. Biol.* **41**, 186–199 (2021).

43. P. Stachon, A. Heidenreich, J. Merz, I. Hilgendorf, D. Wolf, F. Willecke, S. Von Garlen, P. Albrecht, C. Härdtner, N. Ehrat, N. Hoppe, J. Reinöhl, C. Von Zur Mühlen, C. Bode, M. Idzko, A. Zirlik, P2X 7 deficiency blocks lesional inflammasome activity and ameliorates atherosclerosis in mice. *Circulation* **135**, 2524–2533 (2017).

44. L. H. Jiang, A. B. Mackenzie, R. A. North, A. Surprenant, Brilliant Blue G selectively blocks ATP-gated rat $P2X_7$ receptors. *Mol. Pharmacol.* **58**, 82–88 (2000).

45. C. Shen, Y. Zhang, W. Cui, Y. Zhao, D. Sheng, X. Teng, M. Shao, M. Ichikawa, J. Wang, M. Hattori, Structural insights into the allosteric inhibition of P2X4 receptors. *Nat. Commun.* **14**, 6437 (2023).

46. J. Wang, Y. Wang, W. W. Cui, Y. Huang, Y. Yang, Y. Liu, W. S. Zhao, X. Y. Cheng, W. S. Sun, P. Cao, M. X. Zhu, R. Wang, M. Hattori, Y. Yu, Druggable negative allosteric site of P2X3 receptors. *Proc. Natl. Acad. Sci. U.S.A.* **115**, 4939–4944 (2018).

47. A. Karasawa, T. Kawate, Structural basis for subtype-specific inhibition of the P2X7 receptor. *eLife* **5**, (2016).

48. A. C. Oken, N. E. Lisi, I. Krishnamurthy, A. E. McCarthy, M. H. Godsey, A. Glasfeld, S. E. Mansoor, High-affinity agonism at the P2X7 receptor is mediated by three residues outside the orthosteric pocket. *Nat. Commun.* **15**, 6662 (2024).

49. D. W. Nelson, R. J. Gregg, M. E. Kort, A. Perez-Medrano, E. A. Voight, Y. Wang, G. Grayson, M. T. Namovic, D. L. Donnelly-Roberts, W. Niforatos, P. Honore, M. F. Jarvis, C. R. Faltynek, W. A. Carroll, Structure−activity relationship studies on a series of novel, substituted 1-benzyl-5-phenyltetrazole P2X7 antagonists. *J. Med. Chem.* **49**, 3659–3666 (2006).

50. A. Bin Dayel, R. J. Evans, R. Schmid, Mapping the site of action of human P2X7 receptor antagonists AZ11645373, Brilliant Blue G, KN-62, Calmidazolium, and ZINC58368839 to the intersubunit allosteric pocket. *Mol. Pharmacol.* **96**, 355–363 (2019).

51. R. C. Allsopp, S. Dayl, R. Schmid, R. J. Evans, Unique residues in the ATP gated human P2X7 receptor define a novel allosteric binding pocket for the selective antagonist AZ10606120. *Sci. Rep.* **7**, 725 (2017).

52. S. B. de Beer, N. P. E. Vermeulen, C. Oostenbrink, The role of water molecules in computational drug design. *Curr. Top Med. Chem.* **10**, 55–66 (2010).

53. D. Bucher, P. Stouten, N. Triballeau, Shedding light on important waters for drug design: Simulations versus grid-based methods. *J. Chem. Inf. Model.* **58**, 692–699 (2018).

54. CCTU-Core, J. Pharmaceuticals, Cambridgeshire, P. N. F. Trust, *Antidepressant Trial with P2X7 Antagonist JNJ-54175446 (ATP)* (National Center for Biotechnology Information, 2019); https://ClinicalTrials.gov/show/NCT04116606.

55. E. C. Keystone, M. M. Wang, M. Layton, S. Hollis, I. B. McInnes, D. C. S. Team, Clinical evaluation of the efficacy of the P2X7 purinergic receptor antagonist AZD9056 on the signs and symptoms of rheumatoid arthritis in patients with active disease despite treatment with methotrexate or sulphasalazine. *Ann. Rheum. Dis.* **71**, 1630–1635 (2012).

56. C. Chittasupho, Multivalent ligand: Design principle for targeted therapeutic delivery approach. *Ther. Deliv.* **3**, 1171–1187 (2012).

57. A. Arsiwala, A. Castro, S. Frey, M. Stathos, R. S. Kane, Designing multivalent ligands to control biological interactions: From vaccines and cellular effectors to targeted drug delivery. *Chem. Asian J.* **14**, 244–255 (2019).

58. P. J. Reeves, N. Callewaert, R. Contreras, H. G. Khorana, Structure and function in rhodopsin: High-level expression of rhodopsin with restricted and homogeneous N-glycosylation by a tetracycline-inducible N-acetylglucosaminyltransferase I-negative HEK293S stable mammalian cell line. *Proc. Natl. Acad. Sci. U.S.A.* **99**, 13419–13424 (2002).

59. D. N. Mastronarde, Automated electron microscope tomography using robust prediction of specimen movements. *J. Struct. Biol.* **152**, 36–51 (2005).

60. A. Punjani, J. L. Rubinstein, D. J. Fleet, M. A. Brubaker, cryoSPARC: Algorithms for rapid unsupervised cryo-EM structure determination. *Nat. Methods.* **14**, 290–296 (2017).

61. P. Emsley, B. Lohkamp, W. G. Scott, K. Cowtan, Features and development of Coot. *Acta Crystallogr. D Biol. Crystallogr.* **66**, 486–501 (2010).

62. N. W. Moriarty, R. W. Grosse-Kunstleve, P. D. Adams, Electronic Ligand Builder and Optimization Workbench (eLBOW): A tool for ligand coordinate and restraint generation. *Acta Crystallogr. D Biol. Crystallogr.* **65**, 1074–1080 (2009).

63. D. Liebschner, P. V. Afonine, M. L. Baker, G. Bunkoczi, V. B. Chen, T. I. Croll, B. Hintze, L.-W. Hung, S. Jain, A. J. McCoy, N. W. Moriarty, R. D. Oeffner, B. K. Poon, M. G. Prisant, R. J. Read, J. S. Richardson, D. C. Richardson, M. D. Sammito, O. V. Sobolev, D. H. Stockwell, T. C. Terwilliger, A. G. Urzhumtsev, L. L. Videau, C. J. Williams, P. D. Adams, Macromolecular structure determination using X-rays, neutrons and electrons: Recent developments in Phenix. *Acta Crystallogr. D Biol. Crystallogr.* **75**, 861–877 (2019).

64. C. J. Williams, J. J. Headd, N. W. Moriarty, M. G. Prisant, L. L. Videau, L. N. Deis, V. Verma, D. A. Keedy, B. J. Hintze, V. B. Chen, S. Jain, S. M. Lewis, W. B. Arendall Iii, J. Snoeyink, P. D. Adams, S. C. Lovell, J. S. Richardson, D. C. Richardson, MolProbity: More and better reference data for improved all-atom structure validation. *Protein Sci.* **27**, 293–315 (2018).

65. T. Kawate, J. C. Michel, W. T. Birdsong, E. Gouaux, Crystal structure of the ATP-gated $P2X_4$ ion channel in the closed state. *Nature* **460**, 592–598 (2009).

66. X. Liang, D. S. Samways, K. Wolf, E. A. Bowles, J. P. Richards, J. Bruno, S. Dutertre, R. J. DiPaolo, T. M. Egan, Quantifying Ca^{2+} current and permeability in ATP-gated P2X7 receptors. *J. Biol. Chem.* **290**, 7930–7942 (2015).

67. D. S. Samways, B. S. Khakh, S. Dutertre, T. M. Egan, Preferential use of unobstructed lateral portals as the access route to the pore of human ATP-gated ion channels (P2X receptors). *Proc. Natl. Acad. Sci. U.S.A.* **108**, 13800–13805 (2011).

68. D. S. Samways, Z. Li, T. M. Egan, Principles and properties of ion flow in P2X receptors. *Front. Cell. Neurosci.* **8**, 6 (2014).

69. E. C. Meng, T. D. Goddard, E. F. Pettersen, G. S. Couch, Z. J. Pearson, J. H. Morris, T. E. Ferrin, UCSF ChimeraX: Tools for structure building and analysis. *Protein Sci.* **32**, e4792 (2023).

70. R. A. Laskowski, M. B. Swindells, LigPlot+: Multiple ligand–protein interaction diagrams for drug discovery. *J. Chem. Inf. Model.* **51**, 2778–2786 (2011).

71. D. Sehnal, R. Svobodová Vařeková, K. Berka, L. Pravda, V. Navrátilová, P. Banáš, C.-M. Ionescu, M. Otyepka, J. Koča, MOLE 2.0: Advanced approach for analysis of biomacromolecular channels. *J. Cheminform.* **5**, 39 (2013).

72. L. Pravda, D. Sehnal, D. Toušek, V. Navrátilová, V. Bazgier, K. Berka, R. Svobodová Vařeková, J. Koča, M. Otyepka, MOLEonline: A web-based tool for analyzing channels, tunnels and pores (2018 update). *Nucleic Acids Res.* **46**, W368–W373 (2018).
